# Accessibility engineering in web evaluation process: a systematic literature review

**DOI:** 10.1007/s10209-023-00967-2

**Published:** 2023-01-27

**Authors:** Jinat Ara, Cecilia Sik-Lanyi, Arpad Kelemen

**Affiliations:** 1 Department of Electrical Engineering and Information Systems, University of Pannonia, Egyetem u. 10, Veszprem, 8200 Hungary; 2 Department of Organizational Systems and Adult Health, University of Maryland Baltimore, 655 W. Lombard St #455B, Baltimore, MD 21201 USA

**Keywords:** Systematic literature review, Universal inclusion, Digital accessibility, Web accessibility, Engineering assets, Accessibility insights

## Abstract

Several works of literature contributed to the web evaluation process in recent years to promote digital inclusion by addressing several accessibility guidelines, methods, processes, and techniques. Researchers have investigated how the web evaluation process could be facilitated by including accessibility issues to obtain an inclusive and accessible solution to improve the user experience and increase user satisfaction. Three systematic literature reviews (SLRs) have been conducted in the context of past research, considering such research focuses. This paper presents a new SLR approach concerning accessibility in the web evaluation process, considering the period from 2010 to 2021. The review of 92 primary studies showed the contribution of publications on different phases of the web evaluation process mainly by highlighting the significant studies in the framework design and testing process. To the best of our knowledge, this is the first study focused on the web accessibility literature reporting the engineering assets for evaluation of new accessible and inclusive web-based solutions (e.g., websites). Besides, in this study, we aim to provide a new direction to the web designers and developers with an updated view of process, methods, techniques, tools, and other crucial aspects to contribute to the accessible process enrichment, as well as depict the gaps and challenges that may be worthy to be investigated in the future. The findings of this SLR introduce a new dimension in web accessibility research on determining and mitigating the research gap of web accessibility issues for web designers, developers, and other practitioners.

## Introduction

In recent years, various aspects have motivated researchers to conduct studies about digital accessibility. The extension and increased availability of the web for multiple purposes (e.g., information search), the representation of the content (e.g., video, audio), and the emergence of new platforms (e.g., Internet of Things) and technologies (e.g., mobile, computer, tablets) are significant aspects to reinforce the investigation of the digital information platform. In particular, from the very beginning of the digital revolution, digital resources become the fundamental source for citizens to access information such as education, health care, government, news, and other information such as entertainment and sports [1, 2].

According to the World Wide Web Consortium (W3C) and the Web Accessibility Initiative (WAI) report, accessibility is a broad and extensible term associated with people who have disabilities, incompetent skills, or situational-induced impairment [3]. This initiative's objective is to ensure accessibility which means people with special needs should be able to access, navigate, interact, and contribute to the information that is available on the Web/Internet, electronic resources/materials, and computer. The current mission of the WAI initiative is to coordinate international, technical, and human efforts to improve web accessibility [4]. With this mission in mind, WAI launched a set of accessibility guidelines called Web Content Accessibility Guidelines (WCAGs) [5, 6]. A detailed description of WCAG is given in Sect. 2.

The scientific research community has recognized that web design and development must inspect the assorted number of requirements of citizens across the population, including special needs users and elderly citizens. Earlier researchers considered accessibility checking as a supplementary requirement in the evaluation phase of any application development. However, in recent years, researchers suggested that accessibility requirements should be followed from the very beginning of the application design and development. Lack of consideration of accessibility issues during the design and development might introduce violations of accessibility guidelines and consequently basic rights of people with disabilities. A great volume of literature exists addressing accessibility guidelines in the design and development of web platforms [7, 8]. More recently, a few studies highlighted the importance and emerging need of considering accessibility throughout the web development life cycle [9, 10].

Few studies discussed the importance of systematic literature review (SLR) approaches to present the true insights of a particular topic for highlighting future improvement directions [11–13]. Campoverde-Molina et al. [14] mentioned that SLR is a synthesis process of past studies that have been published in different scientific databases focusing on a particular issue. SLR aims to review past literature on a specific domain to determine its effectiveness and find the research gap and new research areas. It helps to identify the way of knowledge improvement, promotes new theories for development, and reveals the new investigated area that needs to focus. Therefore, an SLR focusing on web accessibility engineering assets is essential to determine a way to promote an accessible web platform according to WCAG standards.

Emphasizing the necessity of the SLR approach in the web accessibility context, Akram and Sulaiman [14] and Campoverde‑Molina et al. [15] have conducted SLRs to analyze the accessibility of educational institute websites within a specific period. The first SLR performed the analysis regarding the period between 2009 and 2017. The second one conducted the investigation considering the period of 2009 to 2020. In 2021, Campoverde‑Molina et al. [16] extended their previous work intending to update the result of the past SLR and extended the period from 2002 to 2020. In general, SLR refers to the aggregation of knowledge about a particular domain of research with a set of research questions and solutions. Thus, the SLR process should be as unbiased as possible [17]. The selected SLRs are auditable and have significant effects. However, the focus on engineering assets such as processes, development techniques, and technologies is limited, which is a drawback of SLRs.

This paper presents an extensive SLR in the context of accessibility in the web evaluation process to identify several engineering processes to improve the accessibility of web platforms. This study will help a wide array of people (developers, designers, inventors, leaders, researchers, and users) and facilitate the accessible web design and evaluation process. The paper is organized as follows: In Sect. 2, accessibility concepts, importance, and related works are presented. Section 3 describes the details of conducting the SLR. Section 4 represents the result of conducted SLR and discusses the main findings through a broad discussion. In Sect. 5, we conclude the paper.

## Background and related work

Digital accessibility is a process to ensure the availability of online tools or content to the users [13]. The prime objective of digital accessibility is to make an accessible, operable, and interactable online platform to provide equal information accessing opportunities for people with disabilities [18, 19]. Several aspects might initiate barriers to implementing and ensuring digital accessible platforms or tools or content, such as limited accessibility knowledge and its guidelines. Sometimes organizational barriers and parameters such as organization size, capital, and cost influence accessibility issues. Addressing these issues, the governments and organizations of several countries declared various guidelines, standards, and conformance levels for the stakeholders [20]. Following these guidelines, associate authorities might overcome critical issues and ensure digital accessibility.

### Accessibility standards

To develop an accessible solution (e.g., application, websites, software, etc.), several accessibility guidelines have been introduced by the government of several countries and various public and private institutes such as WCAG, Section 508, EN 301 549, YD/T 1761–2012, WAI-ARIA, BITV, ISO 9241 and ATAG are prominent. Web Content Accessibility Guideline (WCAG) was introduced by the Web Accessibility Initiative of the World Wide Web Consortium with several success criteria under 13 guidelines. Section 508 is accessibility requirements rules published by the US Government for digital resources to make the resources accessible. EN 301 549 is a European accessibility requirement that is suitable for public procurement of ICT products and services in Europe. YD/T 1761–2012 refers to the Chinese Technical requirements standards for web accessibility that primarily focus on ensuring accessibility in the digital platform. Besides, the WAI-ARIA standard was published by W3C to define a set of guidelines for HTML attributes to improve semantic accessibility. BITV is a German standard that is issued focusing on WCAG 2.0 to make the website and application accessible for people with disabilities by ensuring perceivable, operable, understandable, and robust guidelines. Similarly, ISO 9241 provides requirements for accessible developments throughout the application development life cycle. It concerns both hardware and software components for interactive design and development. Authoring Tool Accessibility Guidelines (ATAG) is WCAG and User Agent Accessibility guidelines-based instruction for accessible web content design and development.

Among these guidelines, WCAG is the most widely used accessibility standard. WCAG is a documented guideline that explains all the accessibility criteria and step-by-step recommendations about implementation, improvement, and measurement of accessibility to provide a better user experience, especially for people with disabilities. W3C-based WAI first developed the WCAG standards to make the web accessible [3]. As of July 2022, WAI has published five versions of the WCAG standard, including WCAG 1.0, WCAG 2.0, WCAG 2.1, WCAG 2.2, and WCAG 3.0 (draft version). The WCAG 3.0 is the most sophisticated standard, currently available as a working draft for web developers (front and back end) and designers to develop accessible and usable web content [21].

In 1999, the first version of WCAG 1.0 was released by W3C with three priorities, 14 guidelines, and 65 checkpoints [22]. In 2008, W3C released the second version of standards/guidelines, including 61 success criteria and 12 guidelines under four principles: perceivable, operable, understandable, and robust, concerning three conformance levels: Level A, Level AA, and Level AAA [23]. Furthermore, in 2018, the W3C published an updated version of WCAG 2.0 principles, namely the WCAG 2.1 standard [6]. It has all the principles, guidelines, success criteria, and conformance levels similar to WCAG 2.0 but they added one new guideline and 17 new success criteria. Therefore, completion of the WCAG 2.1 standard ensures the fulfillment of WCAG 2.0 and is followed with more accessibility concerns. The significant update in WCAG 2.1 is the ‘Operable’ principle. In this principle, a new guideline with six success criteria has been added.

**Fig. 1:**
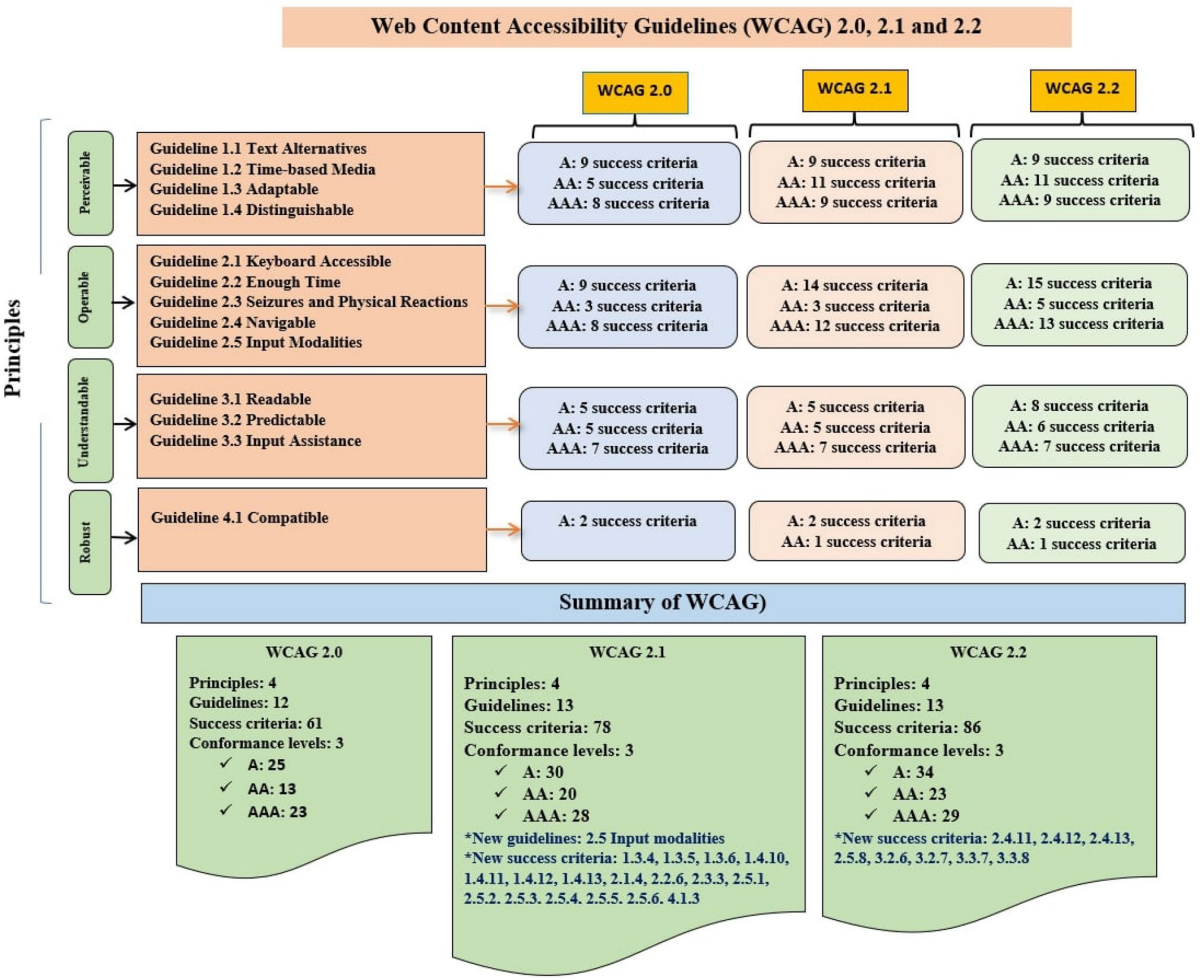
Overview of web content accessibility guidelines (WCAG) version 2.0, 2.1 and 2.2

In 2021, W3C extended the WCAG 2.1 guideline and released the WCAG 2.2, an updated version [24]. In this version, in the Operable principle under guideline (2.4), three new success criteria have been added. In December 2021, the last modified version of WCAG (3.0, working draft) was published, now in progress, waiting for the final draft of guidelines [21]. Figure 1 shows the WCAG standard with its principles, success criteria, and conformance levels. For the details about success criteria and conformance level, the author refers the reader to [24]. In addition, all the versions of WCAG followed three conformance levels of A, AA, and AAA to classify web content. By following the WCAG standard, developers and designers can make digital content accessible for a wide range of people with disabilities, including blindness, low vision or vision impairments, deafness and hearing loss, limited movement, dexterity, speech disabilities, sensory disorders, cognitive and learning disabilities, photo-sensitivity and combinations of these [25]. Nowadays, ensuring an accessible web and improving user experience is crucial for web engineers, researchers, and developers. According to the researchers' opinions, more research needs to be carried out in the next years to improve the accessibility of digital platforms [26]. Therefore, to understand web accessibility in-depth, a detailed and updated SLR approach is important.

Our investigation found seven SLRs between 2010 and 2021 related to the area of digital accessibility (two), web accessibility (three), and web-based image and games accessibility (two). The main focus of these seven SLRs is to make digital content accessible for people with disabilities, which is also a prime objective of the digital accessibility consortium. A detailed discussion of the three SLRs concerning web accessibility has been described in the following subsection (2.2) and a comparison of our SLR with the seven earlier SLR studies is conducted in the discussion section.

### Related SLR studies

In the web accessibility context, the first selected SLR was carried out by Akram et al. [14] to identify the issues with web accessibility of the Saudi Arabian university webpages from the web engineering point of view. To conduct this SLR, they followed three research questions: (1) what are the main principles of Web Content Accessibility Guideline 2.0 (WCAG-2.0) proposed by the W3C to improve web accessibility, (2) what is the compliance level of university and government websites with WCAG-2.0 globally, and (3) what is the compliance level of Saudi Arabian university and government websites with WCAG-2.0. To search past literature, they considered ten scientific databases: Google Scholar, Google search engine, EBSCO host, IEEE Explorer, Science Direct, The Elsevier, Springer Link, ACM Digital Library, Wiley, and Emerald, and found 15 pieces of literature from 2009 to 2017. Their systemic literature review concluded that 87% of the past research employed automatic accessibility testing tools to evaluate university websites. Their SLR also revealed that the most experimented automatic accessibility tools are Bobby, AChecker, eXaminator, TAW, Total Validator, EvalAccess, Cynthia Says, Magenta, Site Analyzer, MAUVE, FAE, WAVE, Valet, and W3C validator service. In addition, they incorporated the manual evaluation process (e.g., interview, questionnaire-based assessment). The manual investigation illustrated that in past research the majority of the work emphasized the improvement of a few accessibility issues such as navigation errors, orientation issues, timing errors, text equivalent to graphics, content, the validity of hypertext markup language (HTML), and cascading style sheets (CSS), use of HTML5, interface design, content, and scripting. However, they conclude that in Saudi Arabia, most universities do not follow World Wide Web Consortium guidelines.

The work proposed by Akram et al. is important in representing the insights of accessibility considering several aspects. However, to validate their represented statistics of implemented automatic accessibility testing with the experimented tools and to identify other possible techniques to validate the accessibility, Campoverde‑Molina et al. [15] carried out the second SLR and present the empirical results of the accessibility evaluation of educational websites. They have considered 25 past studies from 2009 to 2019 to answer ten research questions. This SLR investigated the selected papers focusing on the bibliometric analysis context and literature review. The SLR determined that 80% of past studies focused on automatic analysis through automatic accessibility evaluation tools, 8% through user incorporation, and 12% through hybrid approaches such as expert invitation, user involvement, and automated tools consideration. This SLR concluded that selected websites did not satisfy any version of the WCAG standard and their conformance levels that introduce the necessity of correction of errors by adopting automated tools and manual observation during website construction.

Following their first SLR, Campoverde‑Molina et al. [16] extended their previous SLR considering the period from 2002 to 2020 to investigate more research works to represent the accessibility insights in depth. This recent SLR aimed to analyze past literature that focused on the accessibility analysis of university websites. They performed an investigation of 42 selected papers obtained from three scientific databases (Web of Science, Scopus, and IEEE Xplore), focusing on the accessibility standards and accessibility evaluation methodologies. In 42 papers, they found that 38,416 university webpages have been experimented with in the past years. Their SLR result illustrates that all the experimented websites were from Asia. Most of the existing research has experimented with university homepages. All the past literature followed two standards: ISO/IEC 40,500:2012, and Sect. 508, to analyze the accessibility of web pages. They also concluded that past studies considered automatic evaluation tools to validate university web pages, which is around 90.47%. The most frequently used accessibility testing tools are AChecker, WAVE, Bobby, and TAW. However, the inspection result of this SLR is that most of the past investigated university websites showed violations of accessibility guidelines, most commonly adaptability, compatible, distinguishable, input assistance, keyboard accessible, navigable, predictable, readable, and text alternatives that show important accessibility issues.

The selected three systematic literature reviews represent the current insights of the web in detail, considering the term of accessibility context. Despite the importance of these SLR approaches, they have a poor concern about past research domains and lack consideration of engineering approaches, methods, etc. The lack of engineering methods shows the shortcoming of the past SLR that initiate the importance of a detailed future of SLR. In this paper, our presented SLR is unlike the other three systematic literature reviews. We consider a wide range of existing literature intending to determine the engineering approach to initiate future research to mitigate the current research gap.

##  Research methodology

**Fig. 2:**
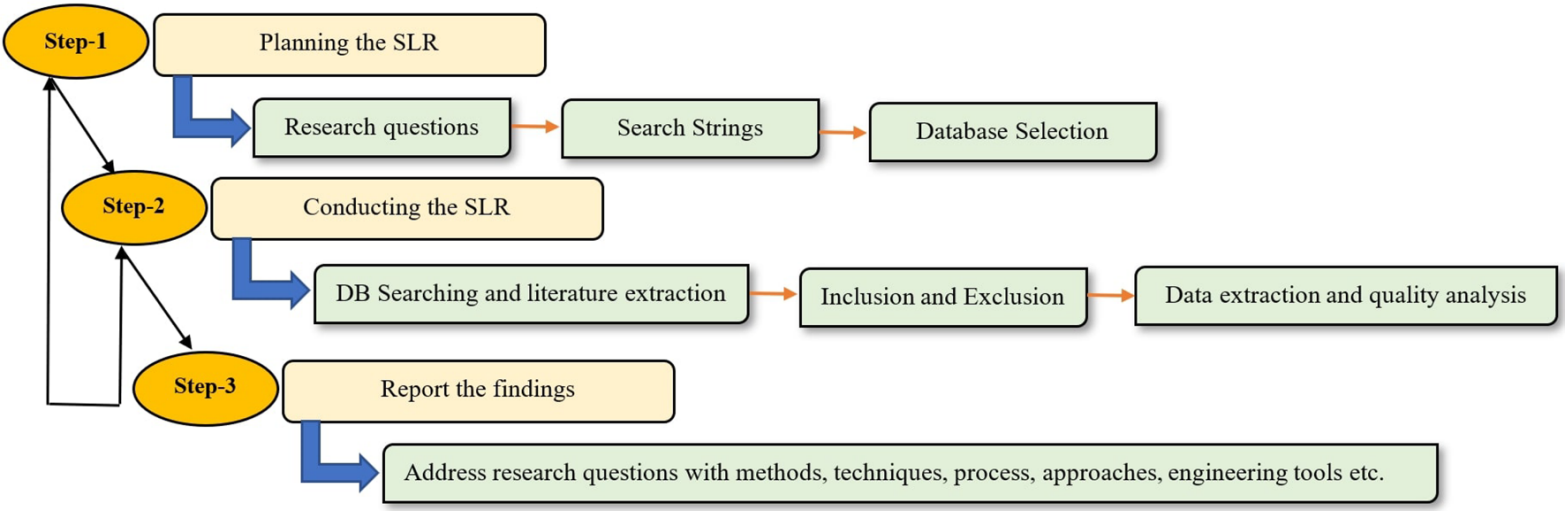
Flowchart of the proposed systematic literature review (SLR)

This study aims to conduct a systematic literature review by following the SLR process guidelines and Kitchenham’s guidelines from Kitchenham and Charters [27]. This research considers three steps to facilitate the SLR approach: (i) planning the SLR process, (ii) conducting the SLR approach, and (iii) reporting the review findings. Figure 2 represents the flowchart of our SLR process.

### Planning the SLR process

The main sub-activities related to planning the SLR are (i) research question specification, (ii) search string formulation, and (iii) database selection. All these sub-activities are described below.

#### Research questions

The first step of a literature review is to develop the research questions. Therefore, we developed the research questions according to our research focus. The two research questions are the following:

**Research Question-1:** What are the available methods, techniques, processes, and approaches to support the evaluation of accessible web?

**Research Question-2:** What are the current engineering assets (tools, technologies, etc.) to support the evaluation of accessible web?

#### Search string

To select the appropriate search strings, we defined a set of keywords according to our research questions concerning the accessibility and website domain. We tested the developed set of keywords in different scientific databases by searching manually and refined it based on the relevancy of the output with the research objective. The finally selected set of keywords represented using Boolean operation is the following:

{(Web engineering) or (Website accessibility) or (Web page accessibility) or (Universal accessibility design) or (Accessibility evaluation) or (Accessibility framework) or (Web accessibility methods and algorithms) or (Accessibility measuring software) or (Current accessibility violations)}.

#### Database selection

For the most relevant and updated literature identification, database selection is crucial. Several scientific databases are available, so appropriate database selection is critical. Herein considering the opinions of other researchers, we selected seven popular databases that provide quality literature and scientific publications. These databases used advanced search algorithms to extract the most related literature according to the user's interest. Seven databases used in this SLR are Scopus, Web of Science, Science Direct, ACM digital library, Google Scholar, IEEE Xplore, and PubMed.

### Conducting the systematic literature review

**Fig. 3:**
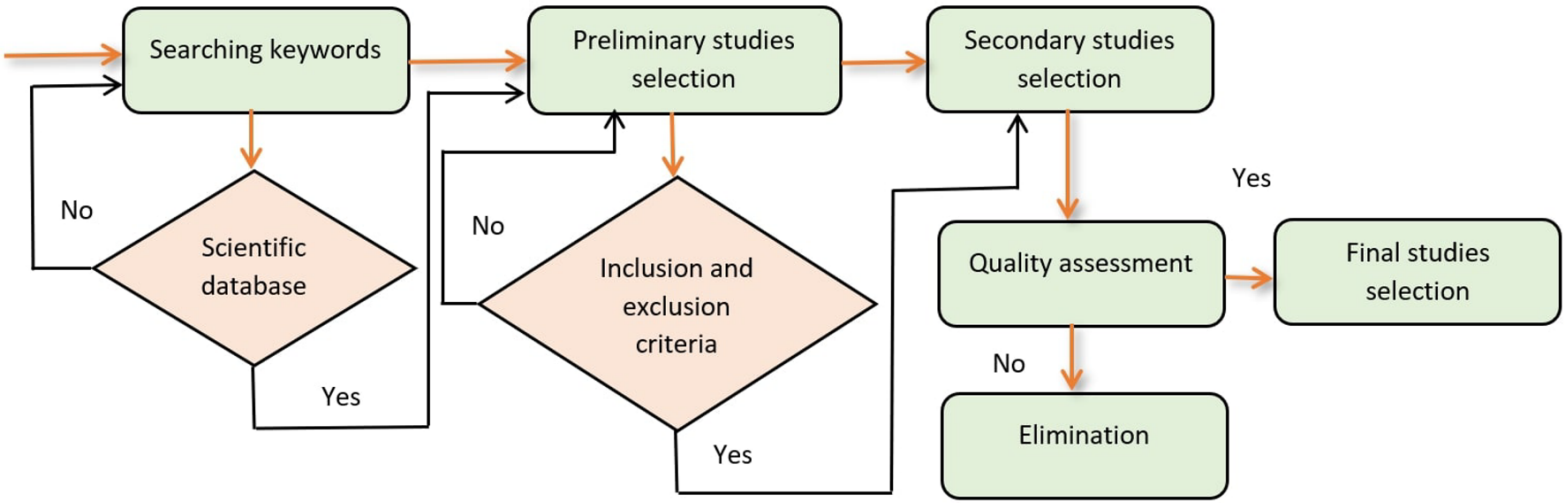
Flowchart of the review overview

This phase aims to describe review activities through the specification of (i) database searching and literature extraction, (ii) inclusion and exclusion implication, and (iii) data extraction and quality assessment. These sub-activities are described in detail in the following subsections. Figure 3 shows the flowchart of the review overview.

#### Database searching and literature extraction

We tested the search strings in seven databases to extract past literature. These databases are accepted by scientific committees for scientific publishing. Most of the literature is open access. These databases have advanced search algorithms and semantic technology to retrieve the appropriate literature according to the search strings.

**Fig. 4:**
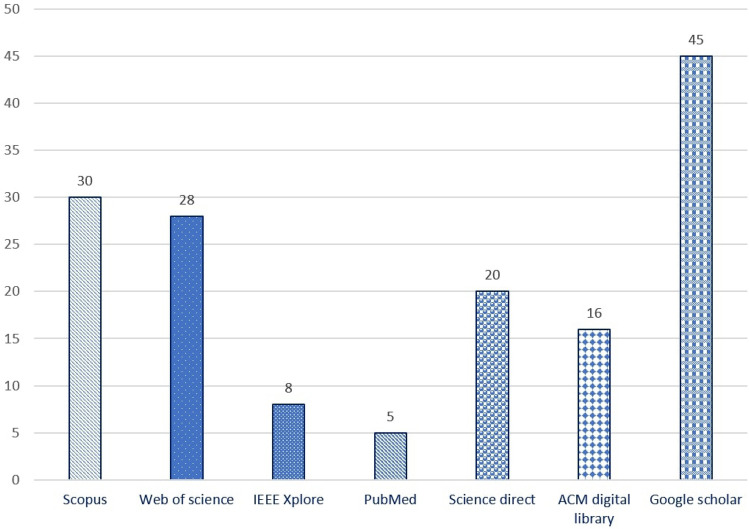
The number of selected literature per database

In total, 152 papers were found in the period from 2010 to November 2021 (Scopus: 30, Web of Science: 28, IEEE Xplore: 8, PubMed: 5, Science directory: 20, ACM digital library: 16 and Google Scholar: 45). Five studies were found from other source and were included in the preliminary screening process. These five papers were found in Research Gate (platform of scientific work) based on the suggestion of digital accessibility expertise (3 papers) and other colleagues’ recommendations (2 papers). These works were not available in the seven databases that we have used in this work. The considered five papers have potential contributions to web accessibility and significant observation that addressed the importance of consideration in this systematic literature review. Figure 4 shows the search result considering the number of papers selected in each database through the search query. However, Scopus, Web of Science, and Google scholar have a wider array of literature than other databases. Among 157 papers, we have selected the most related papers required for this review through inclusion and exclusion criteria (described in the next section).

#### Inclusion and exclusion criteria

The extracted literature has been evaluated to include the most relevant studies in this research. We excluded the literature that did not meet the inclusion criteria for the review. The inclusion criteria were the following: written in English, papers published in peer-reviewed journals or conferences (i.e., not books), publication period between 2010 and 2021, and describe accessibility improvement, development, or related to accessibility assessment.

**Fig. 5:**
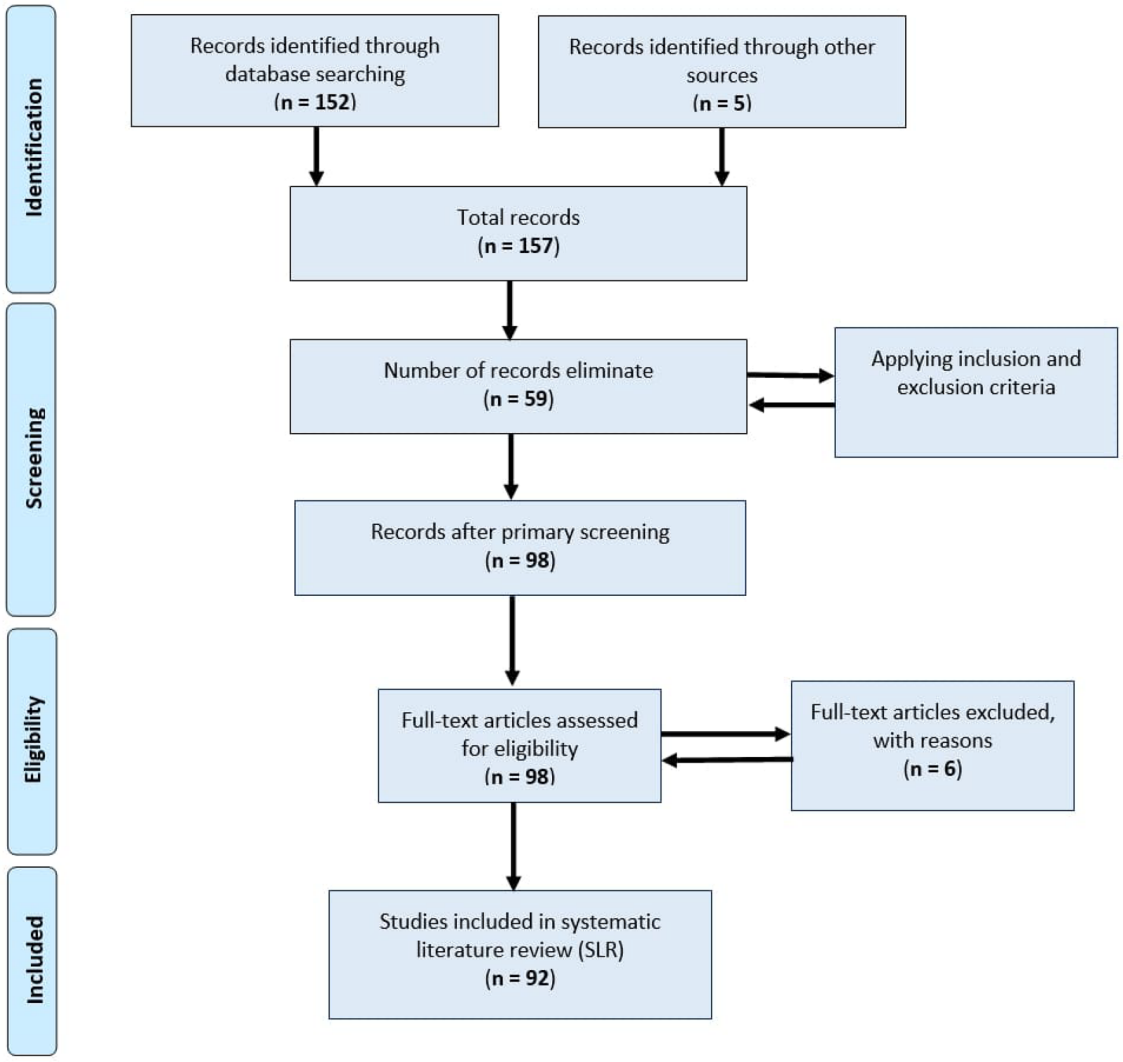
Study selection through PRISMA approach

The exclusion process was performed to eliminate papers from this review. The exclusion criteria were the following: duplicate papers, non-English papers, not directly related or irrelevant papers, papers that are not freely accessible, and those that are not research papers such as posters, letters, thesis, and editorials. After applying inclusion and exclusion criteria to 157 papers, the following observation was made: 12 papers were duplicates, 11 papers were not in English, 29 papers were not directly related to our research focus, and 7 papers were not research papers. In total, we excluded 59 papers by primary screening. After eliminating these papers, we conducted the proposed SLR process considering the selected 98 papers (including 6 past literature reviews). The entire literature selection process has been performed through the Preferred Reporting Items for Systematic Reviews and Meta-Analyses (PRISMA) technique. The PRISMA flow diagram of the study selection is shown in Fig. 5.

#### Data extraction and quality analysis

In this study, our research was conducted based on the search results during 10–15 January 2022, returning 157 papers. To identify a high-quality paper, data extraction and quality assessment are essential. Several earlier literature reviews followed this technique for the primary evaluation of the selected studies. Therefore, we followed assessment guidelines to identify quality papers, complete paper reading, and answer our research questions. Table 1 shows the assessment criteria for the evaluation of selected studies.

**Table 1:** Questionnaire for quality assessments of primary selected studies

Questionnaire for analysis	Options	Output
QA.1: Is the paper a journal article or indexed in SJR or JCR or conference paper?	(+1) Yes/ (+0) No	If yes, rank them?
QA.2: Is web accessibility described in detail in the paper?	(+1) Yes/ (+0) No	If yes, what are the guidelines, principles, and conformance levels?
QA.3: Is web accessibility evaluation software or tool described in the paper?	(+1) Yes/ (+0) No	If yes, which tool is discussed?
QA.4: Is accessibility evaluation and improvement method or engineering approach presented in the paper?	(+1) Yes/ (+0) No	If yes, what method is presented?
QA.5: Does the paper have significant findings?	(+1) Yes/ (+0) No	If yes, what are the findings?

For each question, we set the score to 0 or 1. For each positive answer, a paper gets a score of 1. If not relevant to the assessment questions, the score is 0. For the Q1 indexed journal, the additional points are + 0.50. Similarly, the extra points for the Q2, Q3, and Q4 indexed journal is + 0.40, + 0.30, and + 0.20, respectively. We incorporated Equation-1 and Equation-2 to calculate the final and normalization score to estimate the quality of each selected paper. After conducting the quality analysis, we consider only those studies that passed at least four quality assessment questions with *α* ≥ 0.4 normalized scores. However, among 98 selected studies, six were excluded from this SLR (as shown in Fig. 5, PRISMA diagram) based on the result of the quality assessment criteria. Table 2 shows the quality assessment result of the qualified 92 papers for this review.

1 $$Score_{\left( x \right)} = \sum \left( {QA_{1} + QA_{2} + QA_{3} + QA_{4} + QA_{5} + points} \right)$$

2 $$Normalization = \frac{{Score_{x} - \min \left( {Score_{x} } \right)}}{{\max \left( {Score_{x} } \right) - {\text{min}}\left( {Score_{x} } \right)}}$$

**Table 2:** Quality assessment result of the selected studies

No	Papers	Year	Type of publication		Quality assessment parameter					Normalization		
			Journal	Conference	QA.1	QA.2	QA.3	QA.4	QA.5	Points	Score	Quality
01	Campoverde‑Molina et al. [16]	2021	✓	✗	1	1	1	1	1	0.40	5.40	0.9
02	Yu et al. [30]	2021	✗	✓	1	0	1	1	1	0.00	4.00	0.4
03	Bai [31]	2019	✓	✗	1	1	0	1	1	0.00	4.00	0.4
04	Henry et al. [32]	2014	✗	✓	1	1	0	1	1	0.00	4.00	0.4
05	Wu et al. [33]	2021	✗	✓	1	0	1	1	1	0.00	4.00	0.4
06	Riley-Huff [34]	2012	✓	✗	1	1	1	1	1	0.00	5.00	0.8
07	Marino et al. [35]	2021	✓	✗	0	1	1	1	1	0.00	4.00	0.4
08	Sauer et al. [36]	2020	✓	✗	1	1	1	1	1	0.50	5.50	1.0
09	Vu et al. [37]	2021	✓	✗	1	0	1	1	1	0.30	4.30	0.5
10	Almeida et al. [38]	2010	✗	✓	1	1	1	1	1	0.00	5.00	0.8
11	Gaggi et al. [39]	2021	✗	✓	1	1	1	1	1	0.00	5.00	0.8
12	Acosta-Vargas et al. [40]	2018	✓	✗	1	1	1	1	1	0.50	5.50	1.0
13	Inal et al. [41]	2020	✗	✓	1	1	0	1	1	0.00	4.00	0.4
14	Brajnik et al. [42]	2019	✗	✓	1	1	0	1	1	0.00	4.00	0.4
15	Palaskar et al. [43]	2021	✗	✓	1	1	1	0	1	0.00	4.00	0.4
16	Edelberg et al. [44]	2021	✗	✓	1	1	1	1	1	0.00	5.00	0.8
17	Miesenberger et al. [45]	2019	✗	✓	1	1	1	1	1	0.00	5.00	0.8
18	Alismail et al. [46]	2021	✓	✗	1	1	1	1	1	0.50	5.50	1.0
19	Bhagat et al. [47]	2019	✗	✓	1	1	1	0	1	0.00	4.00	0.4
20	Ojha et al. [48]	2021	✓	✗	1	1	1	1	1	0.50	5.50	1.0
21	Ismail et al. [49]	2018	✓	✗	1	1	1	1	1	0.40	5.40	0.9
22	Kuppusamy et al. [50]	2021	✓	✗	1	1	1	1	1	0.40	5.40	0.9
23	Alshamari [51]	2016	✓	✗	1	1	1	1	1	0.00	5.00	0.8
24	Morris et al. [52]	2018	✗	✓	1	1	1	1	1	0.00	5.00	0.8
25	Alahmadi [53]	2017	✗	✓	1	1	1	1	1	0.00	5.00	0.8
26	Kaur et al. [54]	2021	✓	✗	1	1	1	1	1	0.40	5.40	0.9
27	Hassouna et al. [55]	2017	✗	✓	1	1	1	1	1	0.00	5.00	0.8
28	Kourtiche et al. [57]	2020	✓	✗	1	1	1	1	1	0.00	5.00	0.8
29	Fayzrakhmanov et al. [58]	2010	✗	✓	1	1	1	1	1	0.00	5.00	0.8
30	Li et al. [59]	2016	✗	✓	1	1	1	1	1	0.00	5.00	0.8
31	Alsaeedi [60]	2020	✓	✗	1	1	1	1	1	0.30	5.30	0.9
32	Song et al. [61]	2018	✓	✗	1	1	1	1	1	0.30	5.30	0.9
33	Giovanna et al. [62]	2020	✓	✗	1	1	1	1	1	0.30	5.30	0.9
34	Zeleke [63]	2020	✗	✓	1	1	1	1	1	0.00	5.00	0.8
35	Sanchez-Gordon et al. [64]	2017	✗	✓	1	1	1	1	1	0.00	5.00	0.8
36	Song et al. [65]	2018	✗	✓	1	1	1	1	0	0.00	4.00	0.4
37	Acosta-Vargas et al. [66]	2019	✓	✗	1	1	1	1	1	0.50	5.50	1.0
38	Won [67]	2021	✓	✗	1	1	1	1	1	0.30	5.30	0.9
39	Mohamad et al. [68]	2018	✗	✓	1	1	1	1	1	0.00	5.00	0.8
40	Li et al. [69]	2017	✗	✓	1	1	1	1	1	0.00	5.00	0.8
41	Žuliček et al. [70]	2021	✗	✓	1	1	1	1	1	0.00	5.00	0.8
42	Oliveira et al. [71]	2020	✗	✓	1	1	1	1	1	0.00	5.00	0.8
43	Rashida et al. [72]	2021	✓	✗	1	1	1	1	1	0.30	5.30	0.9
44	Lim et al. [73]	2020	✗	✓	1	1	1	1	1	0.00	5.00	0.8
45	Duarte et al. [74]	2018	✗	✓	1	1	1	1	1	0.00	5.00	0.8
46	Wu et al. [75]	2018	✗	✓	1	0	1	1	1	0.00	4.00	0.4
47	Morato et al. [76]	2021	✓	✗	1	1	1	1	1	0.00	5.00	0.8
48	Boyalakuntla et al. [77]	2021	✓	✗	1	1	1	1	1	0.00	5.00	0.8
49	Michailidou et al. [78]	2021	✓	✗	1	1	1	1	1	0.40	5.40	0.9
50	Bonacin et al. [79]	2021	✓	✗	1	1	1	1	1	0.40	5.40	0.9
51	Antonelli et al. [80]	2018	✓	✗	1	1	1	1	1	0.30	5.30	0.9
52	Csontos et al. [81]	2020	✓	✗	1	1	1	1	1	0.40	5.40	0.9
53	Matoševi´c et al. [82]	2021	✓	✗	1	1	1	1	1	0.40	5.40	0.9
54	Martins et al. [83]	2017	✓	✗	1	1	1	1	1	0.50	5.50	1.0
55	Padure et al. [84]	2020	✓	✗	1	1	1	1	1	0.00	5.00	0.8
56	Hassouna et al. [85]	2015	✗	✓	1	1	1	1	1	0.00	5.00	0.8
57	Pribeanu et al. [86]	2011	✓	✗	1	1	1	1	1	0.00	5.00	0.8
58	Verkijika et al. [87]	2020	✓	✗	1	1	1	1	1	0.40	5.40	0.9
59	AlMeraj et al. [88]	2021	✓	✗	1	1	1	1	1	0.40	5.40	0.9
60	Sharma et al. [89]	2021	✓	✗	1	1	1	1	1	0.30	5.30	0.9
61	Abduganiev [90]	2017	✓	✗	1	1	1	1	1	0.00	5.00	0.8
62	Chapman et al. [91]	2019	✓	✗	1	1	1	1	1	0.40	5.40	0.9
63	Rysavy et al. [92]	2020	✓	✗	1	1	1	1	1	0.50	5.50	1.0
64	Akgül [93]	2021	✓	✗	1	1	1	1	1	0.40	5.40	0.9
65	Doush et al. [94]	2019	✓	✗	1	1	1	1	1	0.20	5.20	0.8
66	Baule [95]	2020	✓	✗	1	1	1	1	1	0.40	5.40	0.9
67	Ajuji et al. [96]	2021	✓	✗	1	1	1	1	0	0.00	4.00	0.4
68	Grant et al. [97]	2021	✗	✓	1	1	1	1	0	0.00	4.00	0.4
69	Kumar et al. [98]	2021	✓	✗	1	1	1	1	1	0.20	5.20	0.8
70	Burkard et al. [99]	2021	✓	✗	1	1	1	1	1	0.00	5.00	0.8
71	Jo et al. [100]	2022	✗	✓	1	1	1	0	1	0.00	4.00	0.4
72	Eusébio et al. [101]	2021	✓	✗	1	1	1	1	1	0.40	5.40	0.9
73	Kous et al. [102]	2021	✓	✗	1	1	1	1	1	0.40	5.40	0.9
74	Ali [103]	2021	✓	✗	1	1	1	1	1	0.20	5.20	0.8
75	Zare et al. [104]	2021	✓	✗	1	1	1	1	1	0.50	5.50	1.0
76	Król [105]	2021	✓	✗	1	1	1	1	1	0.40	5.40	0.9
77	Yi [106]	2020	✓	✗	1	1	1	1	1	0.40	5.40	0.9
78	Moreno et al. [107]	2021	✓	✗	1	1	1	1	1	0.40	5.40	0.9
79	Krawiee et al. [108]	2017	✓	✗	1	1	1	0	1	0.00	4.00	0.4
80	Grantham et al. [109]	2012	✗	✓	1	1	0	1	1	0.00	4.00	0.4
81	Hadadi [110]	2021	✗	✓	1	0	1	1	1	0.00	4.00	0.4
82	Kimmons [111]	2017	✓	✗	1	1	1	1	1	0.50	5.50	1.0
83	Radcliffe et al. [112]	2021	✓	✗	1	1	1	1	1	0.40	5.40	0.9
84	Sun et al. [113]	2017	✗	✓	1	1	0	1	1	0.00	4.00	0.4
85	Alcaraz Martínez et al. [114]	2021	✓	✗	1	1	1	1	1	0.40	5.40	0.9
86	Cao et al. [115]	2021	✓	✗	1	1	1	1	1	0.40	5.40	0.9
87	Giraud et al. [116]	2018	✓	✗	1	1	1	1	1	0.40	5.40	0.9
88	Najadat et al. [119]	2021	✓	✗	1	1	1	1	1	0.30	5.30	0.9
89	Muniandy et al. [120]	2017	✓	✗	1	1	1	1	1	0.20	5.20	0.8
90	Baldwin et al. [121]	2021	✓	✗	1	1	1	1	1	0.40	5.40	0.9
91	Oh et al. [122]	2021	✓	✗	1	1	1	1	1	0.40	5.40	0.9
92	Salvador-Ullauri et al. [123]	2020	✓	✗	1	1	1	1	1	0.40	5.40	0.9

### Reporting the findings

In general, selected papers were related to web development, web accessibility, and information and communications technology (ICT) tools. The statistics of past research showed that existing SLRs focused on a few criteria, but other aspects also need to be considered. However, this study focused on previous SLR results and added new findings from our investigation results that were not highlighted in the earlier SLRs. Earlier SLRs considered accessibility requirements, standards, frequent violations, and improvement suggestions. However, accessible development criteria, evaluation tools development and their engineering methods, and updated validation and testing procedure need to highlight to identify the new research area. According to Durdu and Yerlikaya [28], before ensuring accessible web, web developers and designers should consider the standard guidelines and the requirements of people with disabilities. Also, Bradbard and Peters [29] shared the same observation. They highlighted that the majority of developers and designers have no adequate knowledge about accessibility requirements for people with disability and also lack knowledge about accessible web application development. Thus, in recent days, accessibility specialists have suggested checking accessibility criteria during the development and testing process through automatic accessibility testing tools and user and expert testing. Past works introduced various aspects of developing an effective webpage, but recent studies revealed that accessibility issues completely align with user satisfaction or usability. Therefore, the government of different countries and public and private organizations initiated a few guidelines concerning accessibility and usability criteria [30] that directed a new research area to make the development easier and barrier-free. In the Following, we would like to describe our findings and analysis results of the selected literature in the context of two research questions.

**RQ-1: What are the available methods, techniques, processes, and approaches to support the evaluation of accessible web?**

**Fig. 6:**
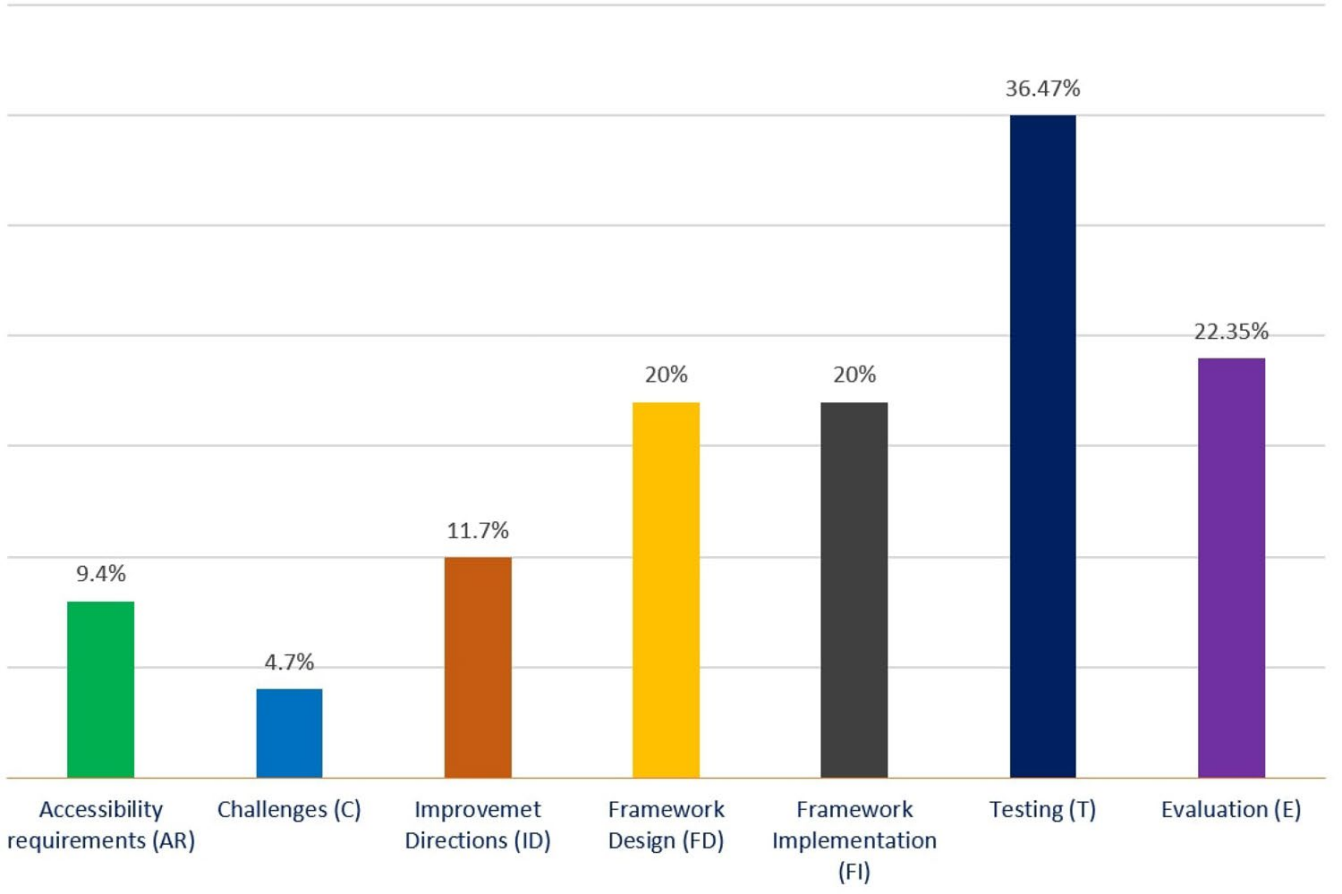
Percentage of studies considering each process related to web evaluation and accessible web applications

**Fig. 7:**
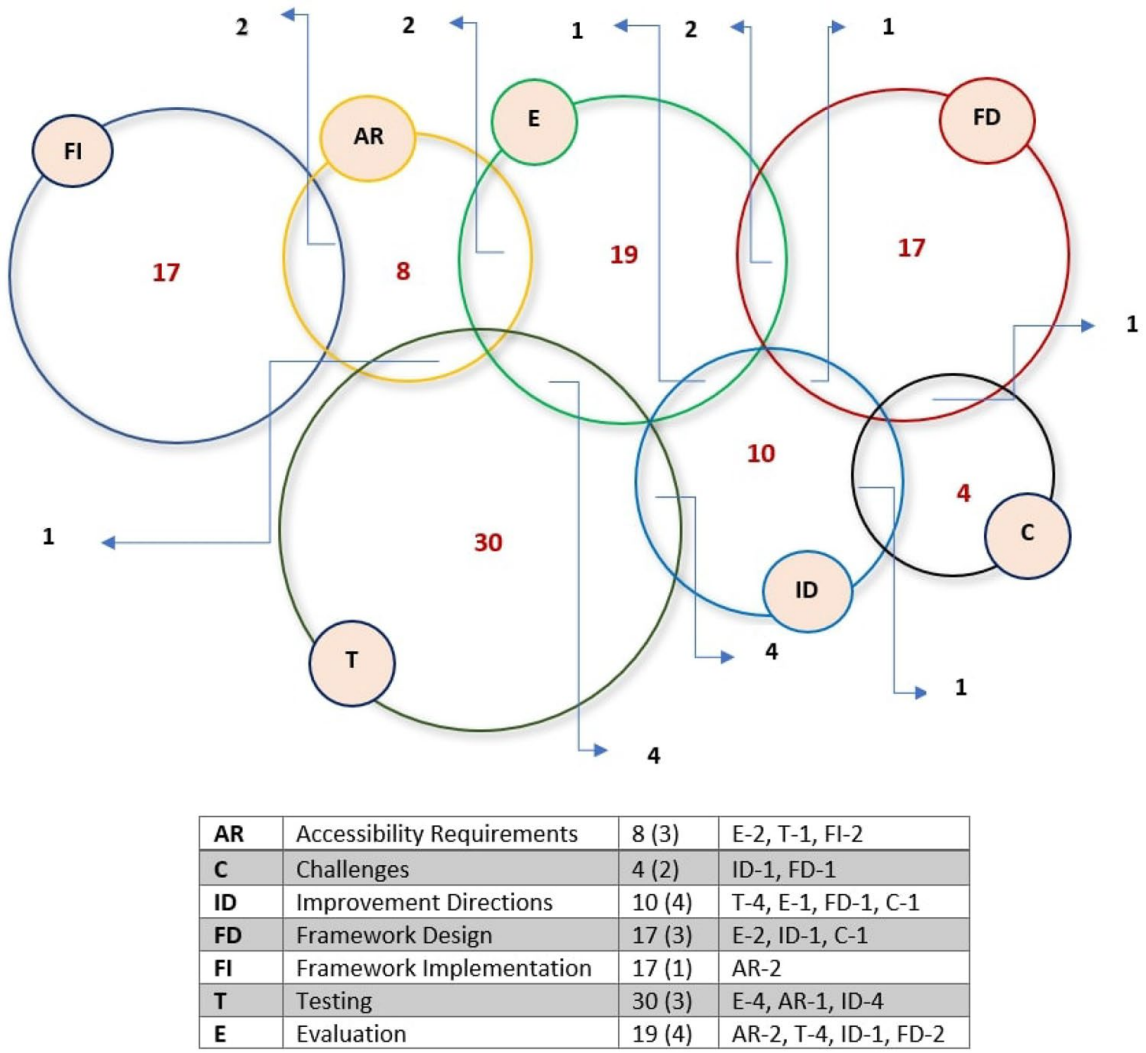
Venn diagram representing the number of studies for certain activities and multiple activities

To answer the first research question, we analyzed 92 selected studies. The selected papers were classified into seven groups/processes: (i) accessibility requirements (AR), (ii) challenges (C), (iii) improvement directions (ID), (iv) framework design (FD), (v) framework implementations (FI), (vi) testing (T), and (vii) evaluation (E). All these phases are described in detail in the following subsections. Figure 6 presents the seven processes with an accounted number of papers for each process. Furthermore, nineteen studies emphasized two activities as presented in the Venn diagram of Fig. 7, which is: {2 (AR & E) + 1 (AR & T) + 2 (AR & FI) + 1 (C & ID) + 1 (C & FD) + 4 (ID & T) + 1 (ID & E) + 1 (ID & FD) + 2 (FD & E) + 4 (T & E)}. The Venn diagram represents the number of papers that have multiple focuses instead of a particular focus or objective. In total 19 unique papers have been found that have multiple focuses. Figure 7 shows the number of papers with their associated activities through the blue arrow. For example, considering ‘accessibility requirements,’ 2 papers focused on accessibility requirements and evaluation process, 2 papers focused on accessibility requirements and implementation, and 1 paper focused on accessibility requirements and testing. Figure 7 shows the complete view of the number of papers with their multi-focused area. Moreover, results depict that past research mostly emphasized the technical processes, especially improvement direction, testing, and evaluation.

####  Accessibility requirements (AR)

This section describes the accessibility and usability requirements with new methods for imposing the accessibility and usability requirements on the current web. Among 92 papers, nine (9) were related to accessibility requirements (representing 9.7% of the total literature) that emphasized ensuring the accessibility guidelines. These studies could be grouped into three main topics of interest, as presented in Table 3.

**Table 3:** The nine studies related to accessibility requirements (AR), grouped by three topics of interest

References	Topic of interest
Bai [31] Henry et al. [32] Wu et al. [33] Riley-Huff [34] Marino and Alfonzo [35]	AR_1_. Importance of accessibility and usability guidelines
Sauer et al. [36]	AR_2_. Accessibility, usability, and user experience improvements methods
Vu et al. [37]	
Almeida and Baranauskas [38] Gaggi and Pederiva [39]	AR_3_. Accessibility requirements specification

In the context of accessibility requirements, Bai [31] and Henry et al. [32] described the importance of accessibility and usability criteria in web and mobile software applications. They added that improving web accessibility is essential for users with disabilities and non-disabled users. They indicate a significant gap between the needed strategies and the developed solutions for people with disabilities, including auditory, cognitive, neurological, physical, speech, and visual impairments. Therefore, the requirements of people with disabilities should be acknowledged during development as accessible technology is essential for equal access and interaction in today's digital world. Also, Riley-Huff [34] pointed out that the first step to developing an accessible website is following the web accessibility guidelines/standards. To ensure higher accessibility standards, a possible way is to improve accessibility and usability [35]. Thus, automatic accessibility testing tools are essential. To ensure usability, they mentioned a few existing models that are prominent to analyze. Another study by Wu et al. [33] investigated data visuality (chart type, chart embellishment, and data continuity) for people with intellectual and developmental disabilities. They emphasized that people with intellectual and developmental disabilities perform information processing differently. But the actual scenario is quite challenging that complicates the process of data visuality for these people. Thereby they suggested considering all the potential requirements with disabilities during development to improve data visuality and accessibility.

Sauer et al. [36] identified three criteria: accessibility, usability, and user experience. These are essential for making the internet platform accessible and convenient for people with and without disabilities. They suggested several methods to ensure accessibility (checklists, cognitive barrier walkthroughs, automatic checking), usability (user testing, observation, questionnaires, interviews, focus groups, heuristic evaluation, cognitive walkthrough, and data logging), and user experience. They suggested that accessibility and usability could be imposed during development to improve the user experience. Vu et al. [37] addressed that low-quality web designs often lead to user frustration that might cause abandonment of undesirable sites. They highlighted several potential and usable web design and evaluation components/methods to improve website usability, which results in a better user experience.

Furthermore, Almeida and Baranauskas [38] pointed out that web accessibility requirements for people with disabilities are crucial. The difficulty of understanding accessibility guidelines is the prime cause of inaccessible design and development. They also added that developers and designers are not experts and have limited knowledge of accessibility requirements. Therefore, they proposed an inclusive web-based collaborative tool to evaluate and modify the guidelines according to the universal and accessible design and development guidelines. It helps to represent the accessibility guidelines more skillfully. Furthermore, Gaggi and Pederiva [39] developed a tool for designers and developers concerning the same issue. They assisted the importance of accessibility measurement with a complete direction about guidelines that need attention during the web design and development phase.

#### Challenges (C)

This section describes the accessibility challenges that are generally liable for the current inaccessible web platform. Among 92 papers, four (4) studies were related to accessibility challenges (representing 4.3% of the total literature). These investigated studies could be grouped into three main topics of interest, as presented in Table 4.

**Table 4:** The four studies related to challenges (C), grouped by three topics of interest

References	Topic of interest
Acosta-Vargas et al. [40] Inal et al. [41]	C_1_. Limited resource adequacy
Brajnik and Vigo [42]	C_2_. Success criteria validation
Palaskar et al. [43]	C_3_. Rules optimization

Researchers are trying to ensure an accessible web for more than a decade, including digital content, websites, user-machine interface, software, etc. Acosta-Vargas et al. [40] pointed out that to implement an accessible web, web researchers have found several challenges. They specified that accessible web page development required adequate knowledge that demands financial investment such as manufacturing and maintaining costs, testing costs, and quality assurance costs. These deliberations are crucial to improving the accessibility of the developed system. However, these deliberations rely on the organization's size, capital, opportunities, etc. Thus, ensuring these necessities is comparatively challenging. Inal et al. [41] showed their effort by conducting a user survey about digital accessibility practices to identify the challenges of creating an accessible system. They invited user experience (UX) professionals to find the most common challenges. The challenges were associated with time constraints, lack of training cost constraints, work overload, not being a requirement for the organization, not being a customer requirement, and people with disabilities or special needs not included as target users. Inal et al. highlighted that such challenges act as barriers to considering accessibility requirements seriously, which is responsible for the current inaccessible web.

Another study by Brajnik and Vigo [42] addressed some crucial challenges that need to consider for introducing an accessible web. The most pressing ones are validity, reliability, sensitivity, and adequacy of user-tailored metrics. Challenges with validity are associated with different validation systems of metrics. For example, there are no specific/gold standards to produce the output for the validation process. The reliance on tools and their limited coverage, completeness, and correctness are heterogonous issues that arise as challenges during metrics result in validation. The reliability of several evaluation performance metrics (human judgment, automatic evaluation, etc.) depends on the evaluation metric transparency and their reproducible and comparable results. Brajnik and Vigo depict that the actual cause for low reliability is the adopted sampling method to evaluate the pages, such as accessibility violation criteria, identified data, formulae, or methods to compute the final score. Sensitivity and adequacy are related to the meaningfulness and suitability of the generated scores through metrics. User-tailored metrics depend on the user's ability as all users have different needs. Accessibility barriers affect different ability users in various manners. Thus, such aspects addressed by Brajnik and Vigo need to be considered in future research.

Furthermore, Palaskar et al. [43] revealed that existing automated accessibility testing tools consider around 50% of Web Content Accessibility Guidelines. Though most of the rules are easy to understand, sometimes it is pretty challenging to implement all the natural language rules in an automatic system. They also claimed that some rules are unacceptable for ensuring accessibility, and others are inappropriate, for example, rules for color schemes and image captions accessibility checking. To develop an accessible website, consideration of some specific aspects is insufficient. Sometimes, accessibility checking requires more than the considered rules. Thus, validating the appropriate rules and incorporating all the guidelines is the major challenge for current web-based accessibility research.

#### Improvement directions (ID)

This section describes directions for future improvement of accessible development. Among 92 papers, ten (10) studies were related to improvement directions (representing 10.8% of the total literature). These investigated studies could be grouped into two main topics of interest, as presented in Table 5.

**Table 5:** The ten studies related to improvement directions (ID), grouped by two topics of interest

References	Topic of interest
Edelberg and Verhulsdonck [44] Brajnik and Vigo [42] Miesenberger et al. [45] Alismail and Chipidza [46]	ID_1_. Technological aspects for accessible development
Bhagat and Joshi [47] Ojha et al. [48] Ismail et al. [49] Kuppusamy and Balaji [50] Alshamari [51] Morris et al. [52]	ID_2_. Technical aspects for accessible prototype design

In the context of improvement direction, few studies focused on the technological aspects of accessible development. Edelberg and Verhulsdonck [44] addressed that web developers and associated authorities choose the colors and font based on the choice of organization identity. However, through this process, it is not always possible to address accessibility issues such as inaccessible color and contrast, and fonts, which makes a difference in design and development for people with disabilities such as a person with low vision. They suggested that colors, fonts, and supporting elements should be perceived correctly during the development phase. Development should be encoded according to the content management system (CMS) to enhance the user experience of a wider audience. Brajnik and Vigo [42] pointed out the significant progress for accessibility metrics in the last decades. However, immaturity is still present in modern development. Thus, future research for further improvements is indicated. Based on their observation, they added a few improvement directions. For example, the implementation should follow Agile, an iterative development model to keep track of accessibility issues. In addition, following the hybrid approach like human judgments through different levels of expertise and users, such as disability type or user, might improve the accessibility of the development. Miesenberger et al. [45] presented some accessibility challenges related to cognitive disability with associated improvement direction. For instance, individual user-centered and personal services-based design and development should ensure. Accessibility requirements should be tested in development cycles with several testing tools (keyboard/mouse logging, eye tracking, etc.). To better usability design, an advanced development framework or platform for R&D should incorporate, and the development should follow the process model (e.g., Waterfall, Iterative, Spiral, Agile, etc.). In addition, Alismail and Chipidza [46] recommended following the WCAG 2.0 and 2.1 guidelines to develop accessible websites by addressing potential accessibility issues. Also, they emphasized user testing by involving people with disabilities, integrating assistive technologies during web accessibility evaluation, incorporating accessibility requirements during design, development, and maintenance phases, and arranging training for web developers and designers to spread accessibility awareness.

In the accessible prototype design context, Bhagat and Joshi [47] presented a few technical recommendations to overcome accessibility challenges. They also mentioned that all the accessibility requirements should be checked and validated by the website's quality assurance (QA) team. Ojha et al. [48] provided a few guidelines based on their detailed study on improving website accessibility with readability. Readability improvement suggestions are related to website structural components such as hyperlinks and image alt-text. These functions should ensure by incorporating the variable weight-based approach for different elements of web pages. Also, website dynamism should be considered in readability score computation to improve the readability in terms of the accessibility of the website. Furthermore, Morris et al. [52] emphasized ensuring alt text of visual content for screen reader users. They have articulated design guidelines for the representation of visual content with prototype design requirements, especially for people with vision impairments, to facilitate and improve visual content accessibility.

#### Framework design (FD)

This section describes several frameworks designed to contribute to the web evaluation process to facilitate web platform accessibility. Among 92 papers, seventeen (17) were related to framework design (representing 18.4% of the total literature). These investigated studies could be grouped into three main topics of interest, as presented in Table 6.

**Table 6:** The seventeen studies related to framework design (FD), grouped by three topics of interest

References	Topic of interest
Alahmadi [53] Kaur and Gupta [54] Hassouna et al. [55] Sapna and Mohanty [56] Kourtiche et al. [57] Fayzrahmanov et al. [58]	FD_1_. Accessible user-centric design practice
Li et al. [59] Alsaeedi [60] Song et al. [61] Giovanna et al. [62] Zeleke [63] Sanchez-Gordon and Luján-Mora [64] Song et al. [65] Palaskar et al. [43] Kuppusamy and Balaji [50] Acosta-Vargas et al. [66]	FD_2_. Web accessibility evaluation
Won [67]	FD_3_. Accessible color design

To contribute to accessible user-centric design, Alahmadi [53] proposed a state-of-the-art framework for web accessibility evaluation to facilitate accessibility measurement and identify accessibility standards errors. The proposed model ensures user-centered design (UCD) based on usability and accessibility guidelines for deaf, visually impaired, and deaf-blindness people. Kaur and Gupta [54] proposed a quality index evaluation framework to evaluate website design to ensure the quality of web design and development. Hassouna et al. [55] addressed some significant issues for users with visual impairment. Concerning the accessibility requirements for users with vision impairment, they designed an accessible web page prototype. Few studies focused on ontology design. For example, Sapna and Mohanty [56] proposed a large-scale test scenario management process using ontology modeling with the help of Web Ontology Language (OWL) to facilitate the software and web development, and testing process by providing faster and more reliable services. Kourtiche et al. [57] designed an ontology of user-profiles considering user disability context to understand various user requirements during accessible web development. Another study proposed by Fayzrahmanov et al. [58] developed a user interface to improve web navigability considering the user requirements with visual impairment.

In the context of web accessibility evaluation, Li et al. [59] designed an interactive web accessibility evaluation system based on the Chinese government guidelines. This framework incorporates automatic tools and human inspection to make evaluation feasible for large web pages. Alsaeedi [60] proposed a novel framework for evaluating the performance of two accessibility testing protocols in webpage evaluation. Song et al. [61] designed a crowdsourcing-based web accessibility evaluation framework to validate against WCAG. It generates the automatic accessibility score of each evaluated webpage according to the weight of each checkpoint. Giovanna et al. [62] developed an open support accessibility evaluation tool to improve automatic accessible support following accessibility conformance testing (ACT) rules. Sanchez-Gordon and Luján-Mora [64] proposed an agile environment-based accessibility evaluation framework to improve evaluation results based on automated tools, simulators, and expert and user-based testing. In further evaluation, Song et al. [65] addressed the complexity of accessibility evaluation methods and the shortage of experts in this field. These aspects make the accessibility evaluation process difficult and reduce their significance. Thus, they proposed a crowdsourcing-based web accessibility evaluation system that uses decision strategies such as the golden set strategy and time-based golden set strategy. Palaskar et al. [43] claimed that most existing Americans with Disabilities (ADA) tools detect only 50% to 60% of accessibility violations because the rules are not understandable. They developed an API to test websites according to the WCAG 2.0 guidelines and A, AA, and AAA conformance level. Additionally, Acosta-Vargas et al. [66] designed a heuristic method to enable accessibility measurement of websites to ensure an accessible and inclusive web platform.

Additionally, Won [67] developed a color tool to understand website color meaning for accessible design practice. The proposed approach can evaluate webpage HTML design prototypes and provide a clear understanding of product-specific colors, cross-cultural color meanings, and color preference. It assists designers in making better color decisions during the design and development phase.

#### Framework implementations (FI)

This section describes several studies that implemented different approaches to contribute to evaluating an accessible web platform. Among 92 papers, seventeen (17) were related to implementation purposes (representing 18.4% of the total literature). These investigated studies could be grouped into three main topics of interest, as presented in Table 7.

**Table 7:** The seventeen studies related to framework implementation (FI), grouped by three topics of interest

References	Topic of interest
Mohamad et al. [68] Li et al. [69] Žuliček et al. [70] Oliveira et al. [71] Rashida et al. [72] Lim et al. [73] Gaggi and Pederiva [39] Duarte et al. [74] Wu et al. [75] Almeida and Baranauskas [38] Morato et al. [76] Boyalakuntla et al. [77]	FI_1_. Web accessibility evaluation system
Michailidou et al. [78] Bonacin et al. [79] Antonelli et al. [80]	FI_2_. Web accessibility evaluation for visually impaired users
Csontos and Heckl [81] Matošević et al. [82]	FI_3_. Accessible prototype improvements

Concerning web accessibility evaluation, many research studies proposed decision support systems, evaluation tools, algorithms, frameworks, models, and interfaces. Mohamad et al. [68] developed a decision support system for large-scale compliance assessment against web accessibility recommendations and legislation. This architecture aims to provide scalable, interoperable, and integrated web accessibility assessment in the context of user-centric design to develop accessible web and mobile applications. Li et al. [69] proposed an EDBA decision support system for website accessibility evaluation at a lower cost. Among other scientific studies, Žuliček et al. [70] developed an accessibility evaluation tool to evaluate the whole webpage, including subpages, to provide a detailed analysis and simplified code refinement. Oliveira et al. [71] developed an accessibility assessment tool to analyze the strength and weaknesses of the website following the Web Content Accessibility Guidelines. Rashida et al. [72] developed an automated web-based tool to identify the quality of academic websites by considering websites' content of information, loading time, and overall performance metrics. Lim et al. [73] proposed an open-source customized automated accessibility testing tool based on the existing Axe accessibility testing engine to scale up the accessibility testing process. Gaggi and Pederiva [39] developed an automatic tool to assist designers and developers in understanding development aspects that should be considered during the development process to introduce an accessible website. In addition, Duarte et al. [74] developed an algorithm to automatically identify the semantic similarity between web content and its textual description in the context of web accessibility evaluation guidelines or rules. Wu et al. [75] developed a semi-supervised regression algorithm involving manual evaluation (webpage sampling) and automatic accessibility testing to generate the overall evaluation result of the website. Almeida and Baranauskas [38] developed a framework following universal design (UD) accessibility guidelines to help designers overcome accessibility barriers in a web-based system. Morato et al. [76] proposed a framework for automatic website accessibility checking in the context of readability through a linguistic characteristics analyzer to identify the best linguistic feature to detect text readability.

In the context of accessibility evaluation for visually impaired people, Michailidou et al. [78] implemented an open-source web accessibility prediction model to predict and visualize the complexity of web pages in the form of a pixelated heat map. Another work proposed by Bonacin et al. [79] developed an adaptive interface focusing on the requirements of Color Vision Deficiency (CVD) people considering automatic recoloring facilities to facilitate the interaction of CVD people with the web.

Additionally, for accessible prototype development, Matošević et al. [82] developed a machine learning algorithms-based expert knowledge system to classify web pages or parts of web pages to improve search engine optimization (SEO) guidelines.

#### Testing (T)

This section describes the studies associated with the testing purpose for accessibility validation of web platforms. Among 92 papers, thirty (30) were related to accessibility testing (32.6% of the total literature). These investigated studies could be grouped into five main topics of interest, as presented in Table 8.

**Table 8:** The thirty studies related to testing (T), grouped by five topics of interest

References	Topic of interest
Martins et al. [83] Padure and Pribeanu [84] Marino and Alfonzo [35] Hassouna et al. [85] Bhagat and Joshi [47] Pribeanu and Fogarassy-Neszly [86] Verkijika and De Wet [87] AlMeraj et al. [88] Sharma [89] Alismail and Chipidza [46] Abduganiev [90] Ismail et al. [49]	T_1_. Automatic detection of accessibility issues
Chapman et al. [91]	T_2_. Content evaluation for osteoarthritis
Rysavy and Michalak [92]	T_3_. Accessibility evaluation for blind users
Akgül [93] Doush and AlMeraj [94] Baule [95] Ajuji et al. [96] Grant et al. [97] Kumar et al. [98] Burkard et al. [99] Jo et al. [100] Eusébio et al. [101] Kous et al. [102] Ali [103] Zare et al. [104] Król et al. [105] Alshamari [51]	T_4_. Accessibility evaluation
Bai [31] Yi [106]	T_5_. Better user experience

In the context of testing, many research studies focused on accessibility testing tools to validate website accessibility considering several disabilities. Few studies tested accessibility issues by incorporating automated accessibility testing tools. Martins et al. [83] tested eHealth websites using a single accessibility testing tool to identify the accessibility issues. Addressing the effectiveness of multiple automatic testing tools, Padure and Pribeanu [84] applied six accessibility evaluation tools to evaluate their selected websites. They suggested that a single testing tool is not enough to identify all the accessibility issues of a website. In other studies, Marino and Alfonzo [35] claimed that automatic tools are inadequate to clarify all the accessibility issues of websites. Thus, further manual observation is required. Therefore, Hassouna et al. [85] initiated a semi-automated evaluation process utilizing an automatic tool and human observation to evaluate design prototypes of websites. The considered evaluation tool is effective as it identifies problems in the design stage. For example, if it detects any error, it redirects to the design stage to show the problem and repair the design problems without modifying the original code. Also, Bhagat and Joshi [47] observed that a lack of awareness regarding assistive technologies and global accessibility standards is responsible for less inclusive and less accessible website design and development. Thus, they conducted the experimental procedure following automatic and user testing to help service providers, government divisions, and ministries ensure maximum accessibility of online platforms. Rysavy and Michalak [92] evaluated the library tools and services in terms of accessibility and usability with open-source tools that emphasized the involvement of blind student workers to validate the resulting transparency.

In contrast, few studies evaluated websites considering several tools and techniques to measure the performance of accessibility, usability, readability, and quality. Akgül [93] evaluated website accessibility, usability, quality, and readability using several tools and techniques. The author employed online open-source tools for accessibility testing and visual and manual inspection for usability testing considering several design standards and Google search results. For quality performance, Akgül incorporated webpage monitoring software considering download time, page size, and objects per website. Finally, evaluated readability, considering text alignment, webpage language, and all-caps text. Grant et al. [97] examined web accessibility and user experience using the hidden code optimization technique. They aim to motivate better web development practices and improve the overall holistic user experience. Ajuji et al. [96] and Kumar et al. [98] proposed two scientific studies. They depict that though the increasing web interactivity is significantly visible, still people with disabilities are finding it difficult to access. They highlighted that website has non-compliant issues against the W3C guidelines. Thus, to evaluate the websites' conformance to the WCAG, Ajuji et al. implemented an automatic accessibility testing tool to evaluate the websites in terms of Perceivable, Operable, Understandable, and Robust. Kumar et al. considered a simulator to visualize the accessibility issues for different types of disabilities. Burkard et al. [99] counteracted that the importance and awareness of digital accessibility are often not recognized during web development. Due to the complexity of the guidelines, people are not motivated to follow them. Therefore, automatic accessibility barrier checking, identifying, and fixing is an important issue. They considered several accessibility monitoring systems to validate the websites and compare tools in the context of completeness and correctness. Also, Alshamari [51] evaluated the accessibility of E-commerce websites through multiple accessibility evaluation tools to generate evaluation reports, locate potential errors, and direct warnings to help in accessible website design and development. Furthermore, Król et al. [105] evaluated the quality of the websites through automatic testing tools considering website performance, SEO quality, website availability, and mobile friendliness.

In the context of better user experience, Bai [31] emphasized accessibility and usability observation as website accessibility and usability are highly correlated. Bai choose the most frequently used automatic conformance testing tools and several usability testing models. Another study proposed by Yi [106] claimed that most websites are not accessible to people with visual impairment, even not readable by the screen reader. This problem happens as websites have too many menus, multiple frames, and a lack of alternative text. Thus, Yi proposed the web accessibility evaluation process using questionnaire-based user testing incorporating people with visual impairment. All the users tested websites using assistive technologies such as screen readers to share their opinions by answering questions about the websites’ accessibility.

#### Evaluation

This section describes several accessibility evaluation methods and techniques. Among 92 papers, nineteen (19) were related to accessibility evaluation (representing 20.6% of the total literature). These investigated studies could be grouped into three main topics of interest, as presented in Table 9.

**Table 9:** The nineteen studies related to evaluation (E), grouped by three topics of interest

References	Topic of interest
Martins et al. [83] Hassouna et al. [85] Moreno et al. [107] Krawiec and Dudycz [108] Hassouna et al. [55] Kous et al. [102] Grantham et al. [109] Hadadi [110] Król et al. [105]	E_1_. Accessibility evaluation methods
Ojha et al. [48] Kimmons [111]	E_2_. Readability evaluation tools and techniques
Radcliffe et al. [112] Sun et al. [113] Giovanna et al. [62] Alcaraz Martínez et al. [114] Cao and Loiacono [115] Bai [31] Giraud et al. [116] Wu et al. [33]	E_3_. Usability evaluation methods

Here, we focus on the studies performed in the context of evaluation purposes. For accessibility evaluation, few studies focused on the questionnaire and expert-based evaluation. Hassouna et al. [55] and Moreno et al. [107] argued that the web is less accessible for people with vision impairments. They utilized questionnaire-based evaluation for accessibility prototypes with the participation of people with visual impairment. For descriptive analysis of the questionnaire result, they used statistical techniques to observe the relationship between the questionnaire items and the dependent variables. Another study by Hadadi [110] stated that designers are not careful about considering the requirements of disabilities such as color blindness. Thus, they overlooked the accessibility criteria to integrate into the design tools. This work evaluated the accessibility of widely used design tools through user feedback. The aim was to increase accessibility awareness and encourage product designers to design and develop an accessible solution. Alcaraz Martínez et al. [114] addressed that several statistical charts on websites are valuable for representing the information. Unfortunately, charts on websites are not accessible for people with low vision and CVD. Thus, they performed a heuristic accessibility evaluation of statistical charts focusing on the needs of people with low vision and CVD to find the usability problems in user interface design. In another study, Giovanna et al. [62] conducted a quantitative and qualitative analysis of user feedback regarding task completion time and computing success rate metric. In addition, some existing literature focused on automatic testing validator performance assessment and effeteness. Krawiec and Dudycz [108] evaluated the performance of automatic accessibility testing validator considering standards, the number of page validation ability, user interface interactivity, software update, free/commercial, etc. This assessment system helps to understand the most effective tool according to the specific requirements. Kous et al. [102] reinforced that several statistical methods using the quantitative data analysis concept are valuable for validating automatic web accessibility testing results. Grantham et al. [109] claimed that low literacy and numeracy skills sometimes affect user access and understanding of the website's content. Following accessibility guidelines and incorporating advanced assessment criteria against international legal accessibility requirements should be considered to ensure an accessible web.

Considering readability, Kimmons [111] claimed that most websites have accessibility issues with content understanding (readability) and structural elements. These issues introduce serious accessibility problems and act as a leading cause of reducing accessibility. Another work is conducted by Sun et al. [113] to assess e-textbooks’ accessibility. They investigated accessibility considering reading time and accuracy to content-related questions. They evaluated experiment results through composite, average, and weighted average scores to examine user experience and performance. However, Ojha et al. [48] addressed a wide array of accessibility and readability evaluation metrics for online content based on machine learning and statistical language modeling techniques.

In the context of usability evaluation, Radcliffe et al. [112] conducted m-Health app evaluation considering accessibility and usability concerns. The evaluation was performed through rapid user-testing and quantifying usability feedback. The user testing result and usability feedback were validated through several standardized evaluation methods for inclusive design requirements specification. Wu et al. [33] addressed that web designers and developers should focus on usability criteria instead of user experience as ensuring usability improves accessibility. Thus, their paper presents several methods and techniques for usability and accessibility evaluation of web design, such as naturalistic observation, participatory evaluation, web-based methods, prototyping, usability inspections, and usability laboratory testing. Giraud et al. [116] indicated that filtering redundant and irrelevant information is crucial for people with visual impairments similar to sighted users to improve the accessibility of the web. Therefore, to improve website usability, some specific needs of users with visual impairment are emerging to consider. They conducted experiments with users with vision impairment to determine the accessibility of web content in terms of filtered or not irrelevant and redundant information. Also, cognitive load, performance, and participants' satisfaction were investigated through the dual-task paradigm.

**Fig. 8:**
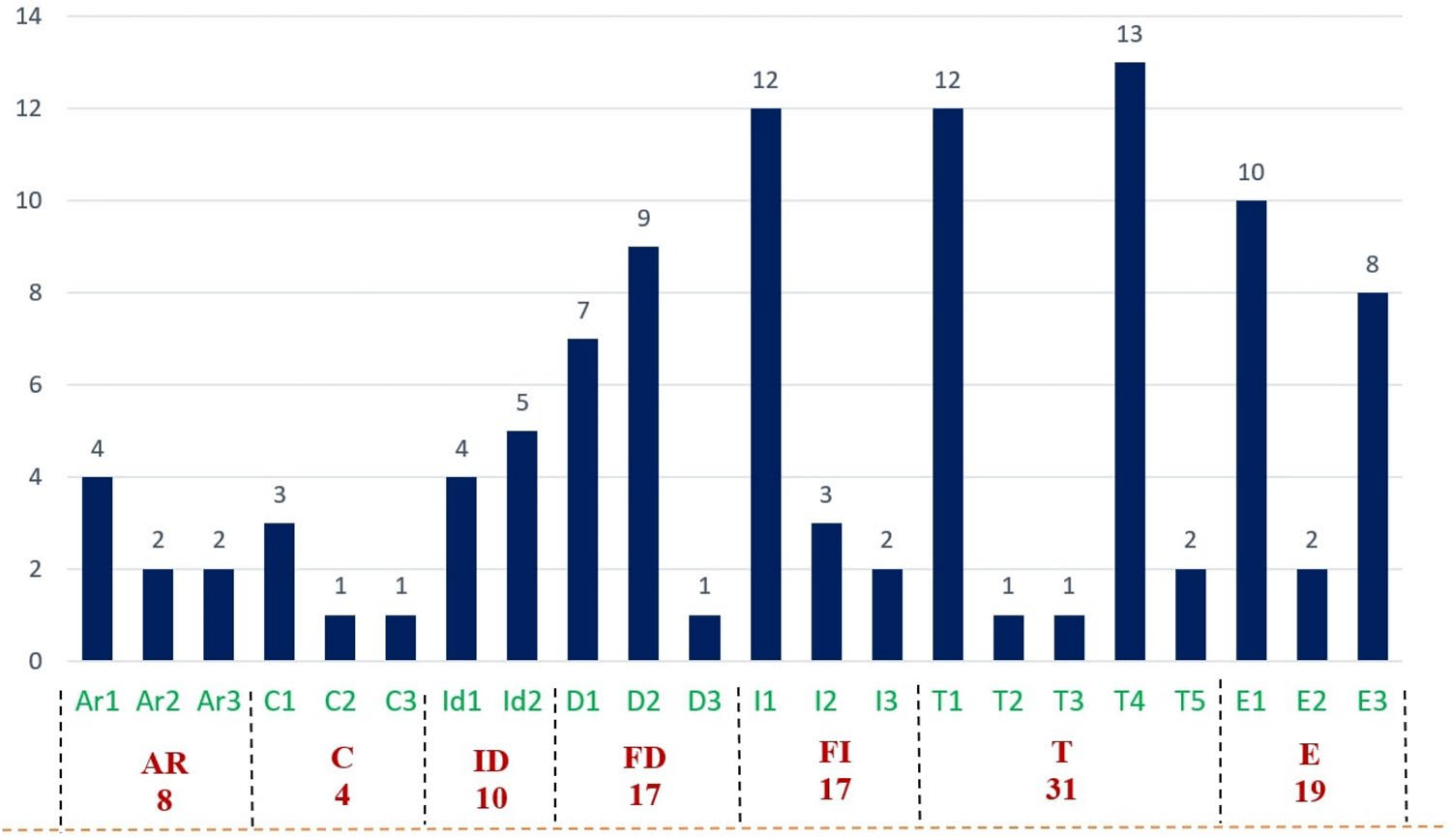
Number of studies on each topic of research interest according to the seven processes/phase

Figure 8 represents the number of papers on each topic of interest according to the seven processes. This figure depicts that the number of proposed approaches for accessibility testing to identify accessibility issues is more frequent than other approaches such as development, implementation, and evaluation. The observation result of research question 1 concludes that the number of the proposed approach for the development and implementation of the accessible web evaluation approach was relatively lower, which addresses a further concern of the web researcher.

**RQ-2: What are the current engineering assets (tools, technologies, etc.) to support the evaluation of accessible web?**

We analyzed the selected papers and identified several groups of interest considering our seven processes. Table 10 summarizes the 22 groups of topics of interest related to the seven processes, including verities of methods, tools, and techniques to answer our second research question.

**Table 10:** Distribution of all the groups of the topics of interest in selected papers related to the seven processes

Process	Groups of the main topic of interest
Accessibility requirements (AR)	AR_1_. Importance of accessibility and usability guidelines
	AR_2_. Accessibility, usability, and user experience improvements methods
	AR_3_. Accessibility requirements specification
Challenges (C)	C_1_. Limited resource adequacy
	C_2_. Success criteria validation
	C_3_. Rules optimization
Improvement directions (ID)	ID_1_. Technological aspects
	ID_2_. Accessible prototype design
Framework design (FD)	FD_1_. Accessible user-centric design practice
	FD_2_. Web accessibility evaluation
	FD_3_. Accessible color design
Framework implementation (FI)	FI_1_. Web accessibility evaluation system
	FI_2_. Web accessibility evaluation for users with visual impairment
	FI_3_. Accessible prototype improvements
Testing (T)	T_1_. Automatic detection of accessibility issues
	T_2_. Content evaluation for osteoarthritis
	T_3_. Accessibility evaluation for the blind user
	T_4_. Accessibility evaluation
	T_5_. Better user experience
Evaluation (E)	E_1_. Accessibility evaluation methods
	E_2_. Readability evaluation tools and techniques
	E_3_. Usability evaluation methods

**Asset description**

From the 7 process groups and 22 topics of interest (Table 10), we aimed to highlight the main assets related to the engineering aspects to support the technical process we have found in our SLR offered by past researchers. These findings will help developers, web engineers, accessibility researchers, and associated authorities to support the accessible design and development process. The addressed assets are listed and described below.

**Assets of accessibility requirements (AR)**

**(AR**_**1**_**.) Assets for the importance of accessibility and usability guidelines:** (1) *explanation* of higher accessibility standards in website evaluation [31]; (2) *explanation* of the importance of accessibility guidelines and user requirements for people with disabilities [32]; (3) sets of usability *requirements* for conventional visualization elements design for cognitive barriers people [33]; (4) *explanation* of web usability and accessibility requirements of WCAG 2.0 and ISO 9241 standards [34].

Therefore, in this group, we identified two subgroups of assets: s-group-1: 3 studies for the explanation, and s-group-2: 1 study for requirements.

**(AR**_**2**_**.) Assets for accessibility, usability, and user experience improvement methods:** (1) *methods* to improve accessibility, usability, and user experience [36]; (2) *methods* to understand user perception to improve usability and user experience [37].

Therefore, in this group, we identified one subgroup of assets: s-group-1: 2 studies for methods.

**(AR**_**3**_**.) Assets for accessibility requirements specification:** (1) Faware is a *framework* for accessibility requirements representation and implementation in visualization elements design and development [38]; (2) WCAG4All is a *tool* for understanding accessibility requirements following standards guidelines [39];

Therefore, in this group, we identified two subgroups of assets: s-group-1: 1 study for framework and s-group-2: 1 study for tools.

**Assets of challenges (C)**

**(C**_**1**_**.) Assets of limited resource adequacy:**(1) *cost* for maintaining, testing, and quality assurance is challenging that depends on organization size, capital, and opportunities [40]; (2) *opportunities* for the training program, learning materials, etc. are not enough for accessibility knowledge improvement [56]; 3) practical *experience and advanced knowledge* of UX professionals from different countries are limited [41].

Therefore, in this group, we identified three subgroups of assets: s-group-1: 1 study for cost, s-group-2: 1 study for opportunities, and s-group-3: 1 study for experience and knowledge.

**(C**_**2**_**.) Assets of success criteria validation:** (1) *metrics* for accessibility evaluation concerning validity, reliability, sensitivity, and adequacy are challenging to ensure [42].

Therefore, in this group, one subgroup of assets was found: s-group-1: 1 study for metrics.

**(C**_**3**_**.) Assets of rules optimization**: (1) *guidelines* are not enough or appropriate, even difficult to incorporate in automated systems or web development processes [43].

Therefore, we found one subgroup of assets in this group: s-group-1: 1 study for guidelines.

**Assets of improvement directions (ID)**

**(ID**_**1**_**.) Assets for technological aspects:** (1) guidelines for accessible and functional prototype design and development [44]; (2) directions for accessible development [42]; (3) directions for cognitive disabilities and their particular accessibility barriers in recent development [45]; (4) suggestions for development that would be facilitated and tested during the design and development phase [46].

Therefore, in this group, we identified three subgroups of assets: s-group-1: 1 study for guidelines, s-group-2: 2 studies for directions, and s-group-3: 1 study for suggestions.

**(ID**_**2**_**.) Assets for accessible prototype design:** (1) *directions* for accessible prototype design and development [47]; (2) *guidelines* for improving website readability by ensuring proper structural components and website dynamism [48]; (3) *suggestions* for spreading awareness, organizing training, and focusing on the accessible prototype design to make the websites accessible to all, including people with special needs [49]; (4) *suggestions* for accessible prototype design to ensure advanced multimedia components [50]; (5) *suggestions* for some potential features that should be taken into consideration during feature development [51]; (6) *guidelines* for visual content representation and accessible prototype design for screen reader users [52].

Therefore, in this group, we identified three subgroups of assets: s-group-1: 2 studies for guidelines, s-group-2: 1 study for directions, and s-group-3: 3 studies for suggestions.

**Assets of framework design (FD)**

**(FD**_**1**_**.) Assets for accessible user-centric design practice:** (1) state-of-the-art *framework* for university website accessibility evaluation for students with hearing and visual impairment [53]; (2) *evaluation* of web page prototype design considering blind user requirement [55]; (3) OUPIP is a user profile-based ontological *model* for designers and developers to develop applications, and devices considering user’s needs, disability type and dynamic context [57]; (4) multi-axial serialization *framework* for the users with visual impairment to understand and find the required information in the webpage [58]; (5) *Ontology* for test management process to provide detailed knowledge about the specific domain and captured requirements for testing [56]; (6) *tool* for quantitative measurement by evaluating website HTML code to identify the quality of the website design [54].

Therefore, in this group, we identified five subgroups of assets: s-group-1: 2 studies for framework, s-group-2: 1 study for evaluation, s-group-3: 1 study for models, s-group-4: 1 study for tool, and s-group-5: 1 study for ontology.

**(FD**_**2**_**.) Assets for web accessibility evaluation:** (1) design a cost-effective crowdsourcing *framework* for web accessibility evaluation considering 25 checkpoints and 5 conformance levels [59]; (2) proposed a *framework* in order to evaluate the well-known automatic accessibility tools in terms of webpage accessibility through their proposed measurement metrics [60]; (3) a crowdsourcing *framework* for web accessibility evaluation against web accessibility content guidelines checkpoints [61]; (4) an open and flexible accessibility testing *tool* to support single and multi-page validation [62]; (5) WUAM is a *framework* for websites usability and accessibility evaluation to improve website performance [63]; (6) proposed a *framework* for web accessibility improvement following ISTQB in agile contexts [64]; (7) proposed a crowdsourcing *framework* for website accessibility evaluation to identify the accessibility barriers and determine the overall accessibility level [65]; (8) proposed an *API* based website accessibility testing tool following ADA guidelines to identify the potential errors and violations, even without prior knowledge [43]; (9) proposed a *framework* for website accessibility barrier measurement according to several variable magnitude techniques [50]; 10) proposed a heuristic *method* to determine the level of accessibility of high ranked websites [66].

Therefore, in this group, we identified four subgroups of assets: s-group-1: 7 studies for framework, s-group-2: 1 study for method, s-group-3: 1 study for tool, and s-group-4:1 study for API.

**(FD**_**3**_**.) Assets for accessible color design:** (1) an accessible color suggestions *tool* for designers to improve their color judgment ability and increase their inspiration for accessible design practice [67].

Therefore, in this group, we identified one subgroup of assets: s-group-1: 1 study for tools.

**Assets of framework implementation (FI)**

**(FI**_**1**_**.) Assets for web accessibility evaluation system:** (1) user-centric holistic decision support environment *system* for web and mobile application accessibility evaluation [68]; (2) a cost-effective task assignment-based decision support *system* for web accessibility evaluation [69]; (3) a *module* for automatically analyzing, identifying and solving the accessibility issues [70]; (4) an automated website readability assessment *model* to improve the accessibility and readability of the website [76]; (5) ShoppingForAll is a *tool* for evaluating and identifying the strength and weaknesses of the website in terms of user satisfaction and accessibility criteria [71]; (6) an *algorithm* for semantic similarity improvement of website content from the web accessibility perspective [74]; (7) a *tool* for quality assessment of the university websites by assessing website source code [72]; (8) FAware is a *tool* to provide accessibility issues and available suggestions [38]; (9) a semi-supervised *model* to evaluate and predict website accessibility [75]; (10) an open-source, industry-standard *tool* to addresses the shortcomings of current accessibility testing tools for the local government context [73]; (11) WCAG4All is a *tool* for consulting web designers and developers about accessibility guidelines [39]; (12) WAccess is a browser extension open-source accessibility testing *tool* to evaluate websites against WCAG guidelines [77].

Therefore, in this group, we identified five subgroups of assets: s-group-1: 2 studies for systems, s-group-2: 1 study for modules, s-group-3: 2 studies for models, s-group-4: 6 studies for tools, and s-group-5: 1 study for algorithms.

**(FI**_**2**_**.) Assets for web accessibility evaluation for visually impaired users:** (1) ViCRAM is a *tool* to predict the visual complexity of the web pages associated with accessibility issues for people with visually impaired or low vision people [78]; (2) FAIBOUD is a *framework* to facilitate the interaction of CVD people with the web [79]; (3) proposed an automatic *system* for identifying website drop-down menu widgets [80].

Therefore, in this group, we identified three subgroups of assets: s-group-1: 1 study for tool, s-group-2: 1 study for framework, and s-group-3: 1 study for the system.

**(FI**_**3**_**.) Assets for accessible prototype improvements**: (1) proposed a *method* to improve accessibility issues by modifying faulty code into correct code to make content management system-based websites more accessible [81]; (2) An expert knowledge *system* to detect web page SEO quality [82].

Therefore, in this group, we identified two subgroups of assets: s-group-1: 1 study for method and s-group-2: 1 study for the expert system.

**Assets of testing (T)**

**(T**_**1**_**.) Assets for automatic detection of accessibility issues**: (1) ACCESSWEB is an *automated validator* for accessibility evaluation considering different accessibility guidelines [83]; (2) TAW is an *automated validator* for web pages evaluation against the web content standards [35]; (3) Total Validator is an *automated validator* to validate accessibility against standards guidelines [86]; (4) A semi-automated *process* is to evaluate website design prototypes and repair without modifying the original page code [85]; (5) AChecker and TAW *automated validators* are to validate the accessibility of the website and identify the associated issues that violated accessibility guidelines [87]; (6) automatic testing by AChecker, Total Validator, WAVE, and HTML/CSS/ARIA *automated validators* for evaluation of higher educational institute websites [88]; (7) hybrid accessibility testing *process* with AChecker, WAVE, and aXe automatic accessibility testing tools and JAWS and Non-Visual Desktop Access, two open-source screen reader applications [47]; (8) WAVE is an *automated validator* to indicate accessibility issues and related accessibility features [89]; (9) AChecker, Cynthia Says, Mauve, TAW, Total Validator, and Wave are *automated validators* to identify the accessibility issues and compare their result to understand the effectiveness of the system [84]; (10) AChecker, WAVE, and SortSite are *automated validators* to identify the shortcoming of websites [46]; (11) AChecker, Cynthia Says, EIII Checker, MAUVE, SortSite, TAW, Tenon, and WAVE are *automated validators* to identify the effectiveness of result considering coverage completeness, correctness, specificity, inter-reliability and intra-reliability, validity, efficiency, and capacity [90]; (12) multi-tool accessibility assessment through *automated validators* such as AChecker, Cynthia Says, Tenon, WAVE, Mauve, and Hera to perform a comparative analysis of websites to identify the effective testing tool [49].

Therefore, in this group, we identified two subgroups of assets: s-group-1: 10 studies for the automated validator, and s-group-2: 2 studies for the process.

**(T**_**2**_**.) Assets for content evaluation for osteoarthritis:** (1) SMOG and FOG are two *automated validators* to determine webpage content readability considering informative images and relevant video [91].

Therefore, we identified one subgroup of assets in this group: s-group-1: 1 study for the automated validator.

**(T**_**3**_**.) Assets for accessibility evaluation for blind users:** (1) WAVE is an online *automated validator* for accessibility issues identification of library tools and services for blind users [92].

Therefore, this group identified one subgroup of assets: s-group-1: 1 study for the automated validator.

**(T**_**4**_**.) Assets for accessibility evaluation:** (1) propose a hybrid *evaluation* approach for improving user experience [97]; (2*)* A hybrid *evaluation* process for accessibility, usability, quality and readability testing [93]; (3) A semi-automated evaluation *process* incorporating AChecker, Total Validator, WAVE and expert opinion to examine the webpage code [94]; (4) AChecker is an *automated validator* to analyze education cooperative websites to determine its accessibility considering disabilities [95]; (5) TAW is an *automated validator* to validate websites against the conformance of WCAG 2.0 [96]; (6) A *simulator* for visual, hearing and mobility impairment to visualize the accessibility issue associated with the particular disability [98]; (7) A semi-automated *process* considering axe Monitor, Pope Tech, Siteimprove, ARC with user feedback to validate websites accessibility [99]; (8) WAVE is an *automated validator* to validate the accessibility of COVID-19 vaccine registration portals [100]; (9) accessibility evaluation through comparative *analysis* using automatic accessibility testing protocols and statistical observation [101]; (10) AChecker is an *automated validator* to evaluate website accessibility [102]; (11) AChecker, Cynthia Says, and TAW are *automated validators* to validate website e-accessibility [103]; (12) A comparative *analysis* using Webaccessibility automated accessibility validator and statistical technique to validate the websites against WCAG 2.1 conformance guidelines [104]; (13) Google PageSpeed Insights, Blink Audit Tool, Backlink Checker, WAVE and Bulk are *automated validator* to assess and evaluate website quality [105]; (14) Achecker, TAW, Eval Access, MAUVE and FAE are *automated validators* to identify the accessibility issues of the selected websites [51].

Therefore, in this group, we identified four subgroups of assets: s-group-1: 2 studies for evaluation, s-group-2: 7 studies for the automated validator, s-group-3: 1 study for the simulator, s-group-4: 2 studies for analysis, and s-group-5: 2 studies for the process.

**(T**_**5**_**.) Assets for better user experience:** (1) FAE, Nielsen’s10-item metric, and Baker’s six-dimension are *automated validators* for accessibility and usability testing for better user experience [31]; (2) questionnaire-based user *assessment *to identify the accessibility incompatibility with screen reader application [106].

Therefore, in this group, we identified two subgroups of assets: s-group-1: 1 study for automated validator and s-group-2: 1 study for assessment.

**Assets of evaluation (E)**

**(E**_**1**_**.) Assets for accessibility evaluation methods:** (1) manual *assessment* through assistive technology with users and experts in this field [83]; (2) questionnaire-based *assessment* for people with visual impairment through several data analysis techniques [85]; (3) questionnaire-based *evaluation* for discovering the navigation strategies of low vision people that cause to experience accessibility barriers [107]; (4) automatic *assessment* system to identify the most effective validator for accessibility testing [108]; (5) statistical data *analysis* to validate the reliability of the questionnaire result [55]; (6) quantitative data *analysis* using statistical analysis methods [102]; (7) manual *assessment* criteria for accessibility assessment of Australian private and governmental websites against DDA standards [109]; (8) user *evaluation* of Adobe online design platforms tool with the help of mix panel data analysis [110]; (9) statistical *evaluation* for quality analysis of the websites [105].

Therefore, in this group, we identified three subgroups of assets: s-group-1: 4 studies for assessment, s-group-2: 3 studies for evaluation, and s-group-3: 2 studies for analysis.

**(E**_**2**_**.) Assets for readability evaluation tools and techniques**: (1) *metrics*/*tools* for website content readability measurement to make website content universally accessible [48]; (2) descriptive *evaluation* of university homepage to validate the readability [111].

Therefore, in this group, we identified two subgroups of assets: s-group-1: 1 study for metrics/tool and s-group-2: 1 study for evaluation.

**(E**_**3**_**.) Assets for usability evaluation methods:** (1) statistical *techniques *for usability testing of m-Health application [112]; (2) questionnaire-based *evaluation* for user experience testing [113]; (3) quantitative and qualitative *analysis *considering the user performance, computing task completion time, and correct task completion ratio [62]; (4) quantitative and qualitative *analysis* with statistical measurements to evaluate user perceptions [115]; (5) statistical *analysis* to determine the relationship between web accessibility and usability [31]; (6) user *evaluation* to improve website accessibility and interface usability by reducing the cognitive load of people with blindness [116]; (7) hybrid *evaluation* process to identify the effectiveness of usability and interface design [33]; (8) quantitative and qualitative *analysis* to evaluate user perceptions for interactive user interface design [114].

Therefore, in this group, we identified three subgroups of assets: s-group-1: 1 study for technique, s-group-2: 3 studies for evaluation, and s-group-3: 4 studies for analysis.

**Fig. 9:**
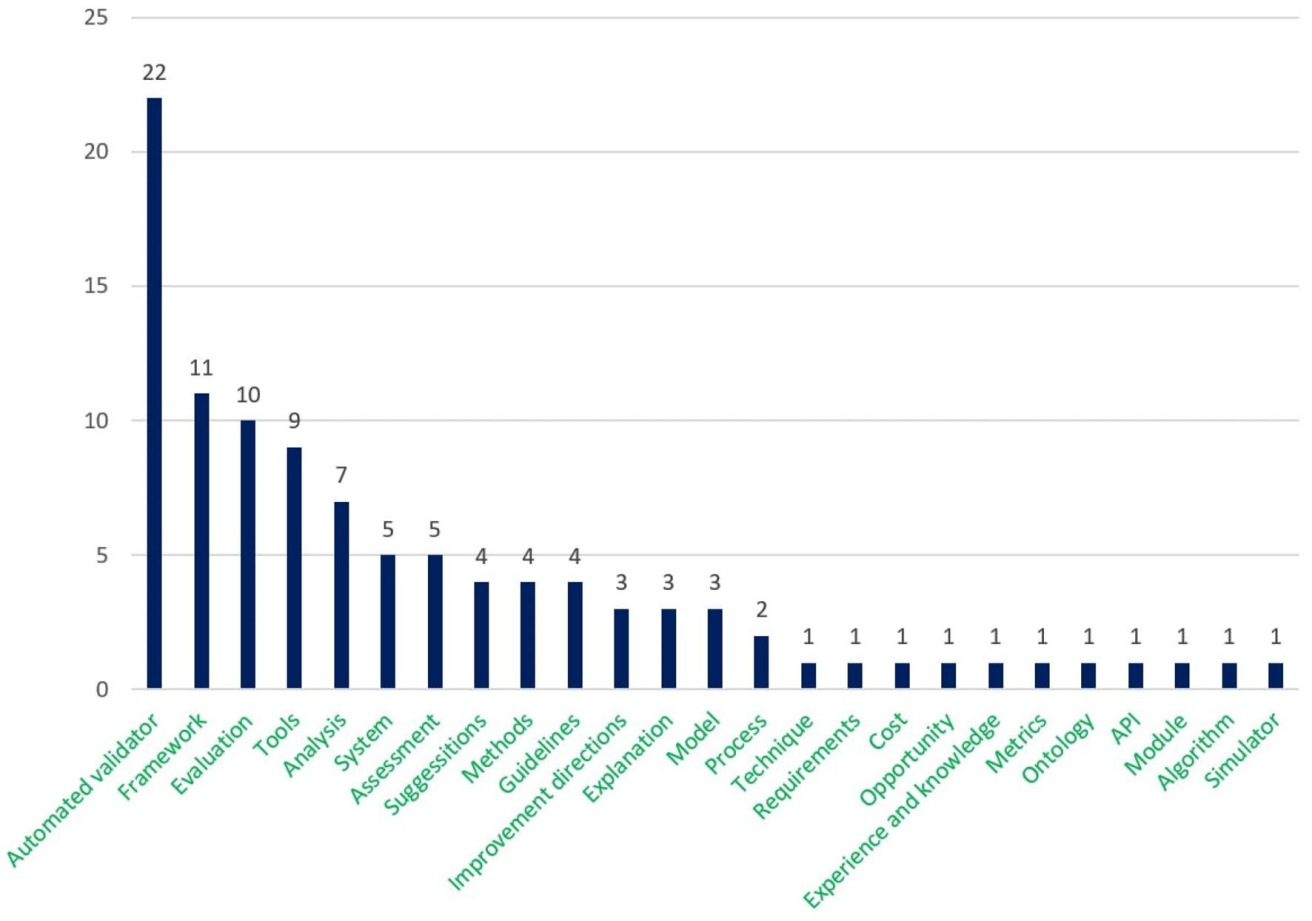
Identified assets of the research outcome

Figure 9 shows the graphical representation of assets obtained in this SLR. The observation result of research question 2 (as shown in Fig. 9) illustrates that automated validators, tools, and frameworks are the main research assets in the investigated area. It demonstrated that most past researchers and the scientific community contributed to accessibility research using the existing automated validators. Recently researchers focused on developing accessibility testing tools and designing frameworks to contribute to accessibility practice, though the number of developed tools and frameworks is limited. In addition, a small group of researchers has conducted studies on other aspects in the accessibility context.

## Discussion

### Research context’s investigation results

**Fig. 10:**
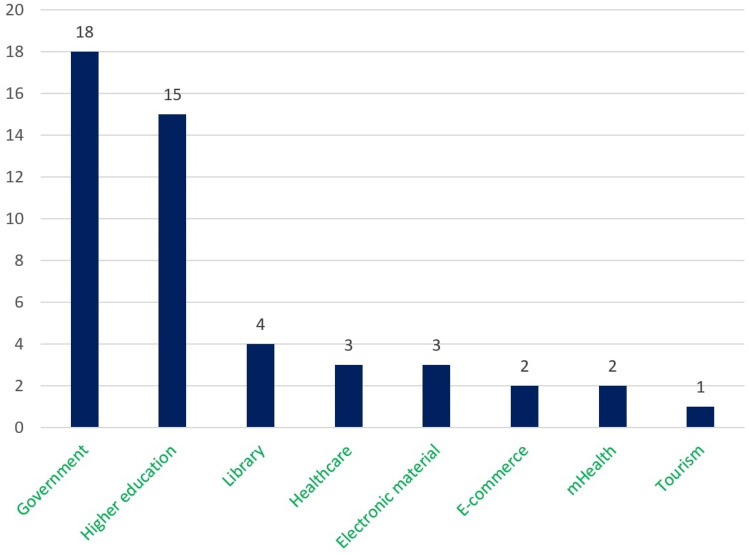
Number of studies of each area of research considering accessibility domain

This section highlights the context we focused on in our investigation to reveal in this SLR. The first context of the discussion is the invested domain of past studies. Figure 10 shows the number of papers in each research area found in this SLR. Most studies focused on education, such as government and higher education institute websites. However, few studies focused on other areas such as libraries, health care, electronic materials (e.g., eBooks, visual charts, etc.), tourism, and E-commerce. Accessibility research has a significant contribution to national and international legislation to develop accessible software or web in different domains. However, more investigation for accessibility measurement should be carried out considering other areas to present accessible systems within a broad scope of future research. Besides, during the COVID-19 pandemic, accessible healthcare websites were significantly valuable and were a crucial requirement for the world community [117]. However, the observation result depicts that the number of proposed studies focusing on the healthcare domain is not adequate, which is the present research gap in this particular domain. This finding exposes the necessity of devoting continued effort to investigating the healthcare domain in future research.

**Fig. 11:**
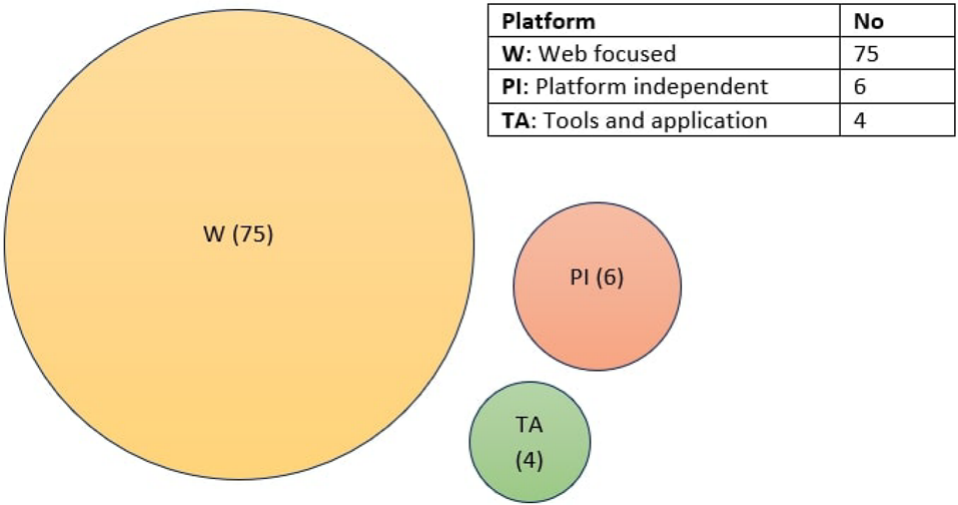
Number of investigated studies of each platform

Figure 11 shows that according to the investigated platforms, most of the selected studies focused on web systems (75 studies), four (4) studies focused on tools and applications, and six (6) studies presented platform-independent approaches.

**Fig. 12:**
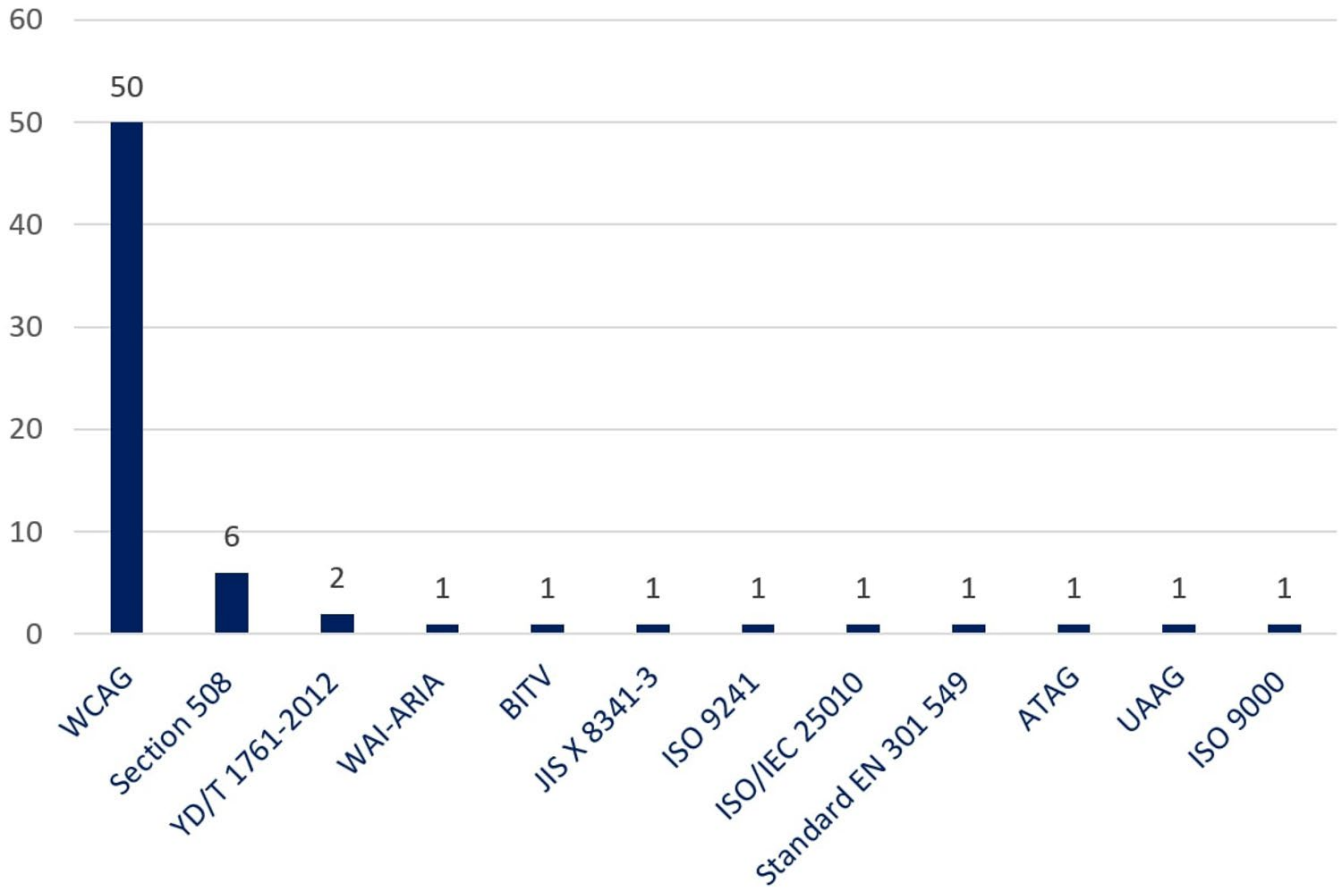
Number of studies according to the focused guideline

Regarding guidelines, most of the selected studies followed WCAG standards to evaluate and develop the web or software application. Figure 12 depicts that WCAG is the dominant and accepted standard for referencing primary accessibility guidelines for the accessible solution and prototype design or user-centric design issues. WCAG is also extensively used as a referencing guideline in accessibility assessment or testing tool development. However, as WCAG is incorporated widely; a few deliberations are laborious to solve by imposing this standard alone. Thus, a wide variety of supporting resources and other guidelines or standards is crucial help for web developers and designers to improve accessibility issues and overcome the current accessibility limitation.

Regarding programming language, the frequently used programming language to implement the proposed methods, tools, and frameworks were JavaScript (object-oriented), Python (high-level programming language), HTML (markup language), CSS/SCSS (style description language), PHP (scripting language), C+ + (case sensitive language), OWL (knowledge representation language) and SWRL (logical inference engine). The most frequently marked engineering tools were Apache and MySQL webserver, Oracle database, JavaScript (React), FontAwesome, Axe, Chrome, and HTML Code Sniffer accessibility evaluation libraries. Frequently applied Application Programming Interfaces (APIs) are Clarifai (for image and video), Indico (for semantic matching), Swoogle, AATT, and REST API for Windows and Linux Operating systems. Most tested websites followed content management systems such as WordPress, Joomla, and Drupal. The tested report represents in extensible markup language (XML), enhanced address recognition logic (EARL), and portable document format (PDF). However, Selenium Web Browser Automation and ChromeDriver Tools with Webdriver and MutationObserver API are effective among other web engineering tools.

**Fig. 13:**
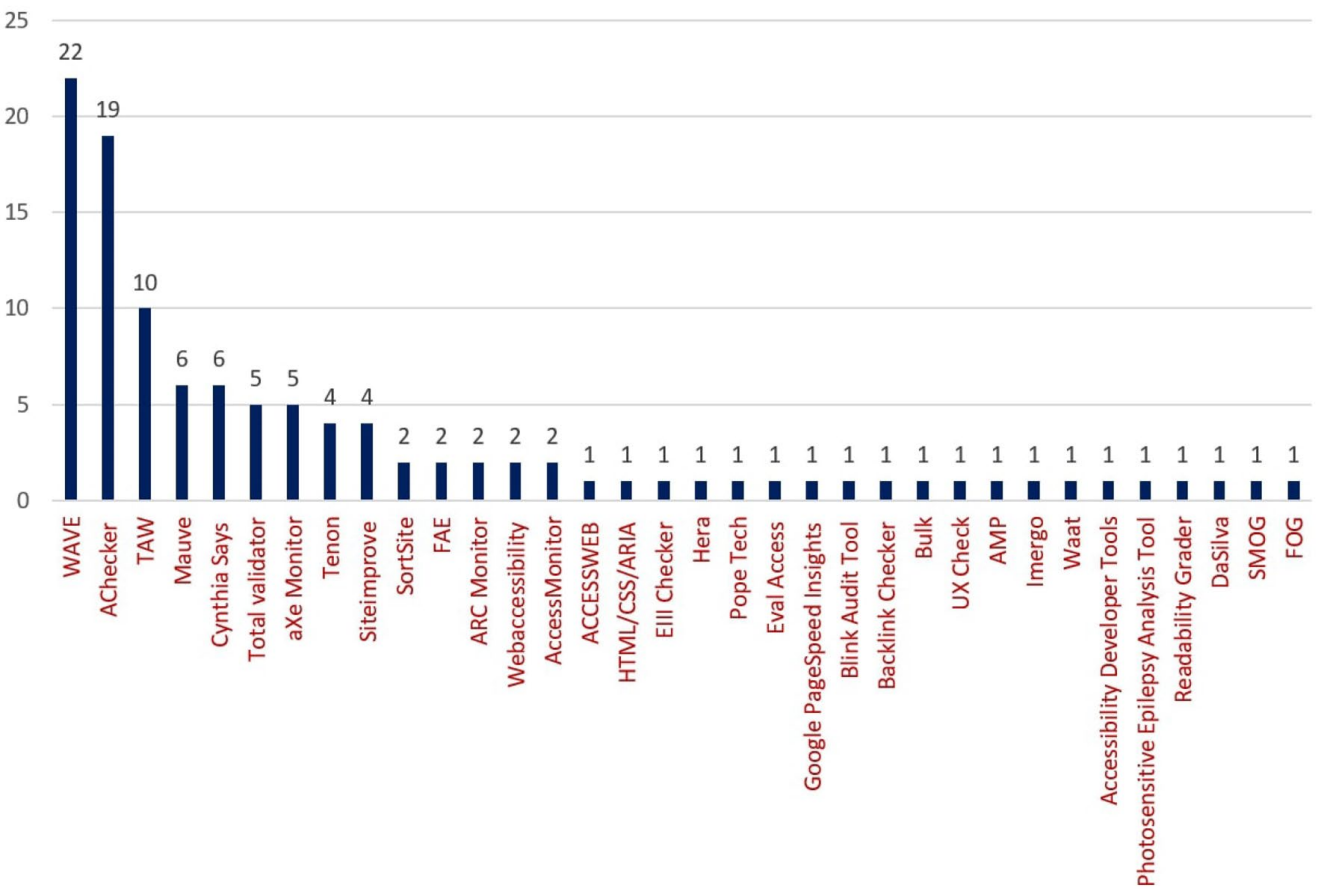
Number of studies considering implemented testing tools

Generally, the effectiveness and performance of the web concerning accessibility issues have been assessed through automatic testing (accessibility and usability) and human observation. Figure 13 shows that frequently implemented testing tools are WAVE, AChecker, and TAW. However, earlier studies also addressed other accessibility and usability testing tools such as Mauve, Cynthia Says, Total Validator, aXe Monitor, Tenon, Siteimprove, SortSite, etc. Among several automatic testing tools, some specific tools have been implemented frequently in the past literature. Despite the availability of a wide array of accessibility testing tools (approximately 75 according to W3C), most tools are underrated, and even web designers and developers have no idea about these tools and their effectiveness [118]. In the investigated works of literature, only three pieces of literature compared multiple automatic accessibility testing tools to evaluate their effectiveness. This limited number of comparative analyses is not sufficient to show the usefulness of the existing automated tools. Thus, it is crucial to devote continued effort to perform further comparative analysis considering the benefits of automatic testing tools in future accessibility research.

Concerning the accessibility and usability evaluation and validation results, SPSS, Microsoft Excel, and STATISTICA were the most used statistical analysis tools. Frequently used statistical standards are standard deviation (SD), Pearson’s correlation analysis, one-way ANOVA, System Usability Scale (SUS), Tierney’s 7-min accessibility assessment and app rating system, z-score calculation, Kolmogorov–Smirnov test, Shapiro–Wilk test, Wilcoxon signed-rank test, arithmetic mean, median, coefficient of variation, minimum and maximum value computation. According to past literature, these statistical techniques are effective in accessibility evaluation and validation practice.

**Fig. 14:**
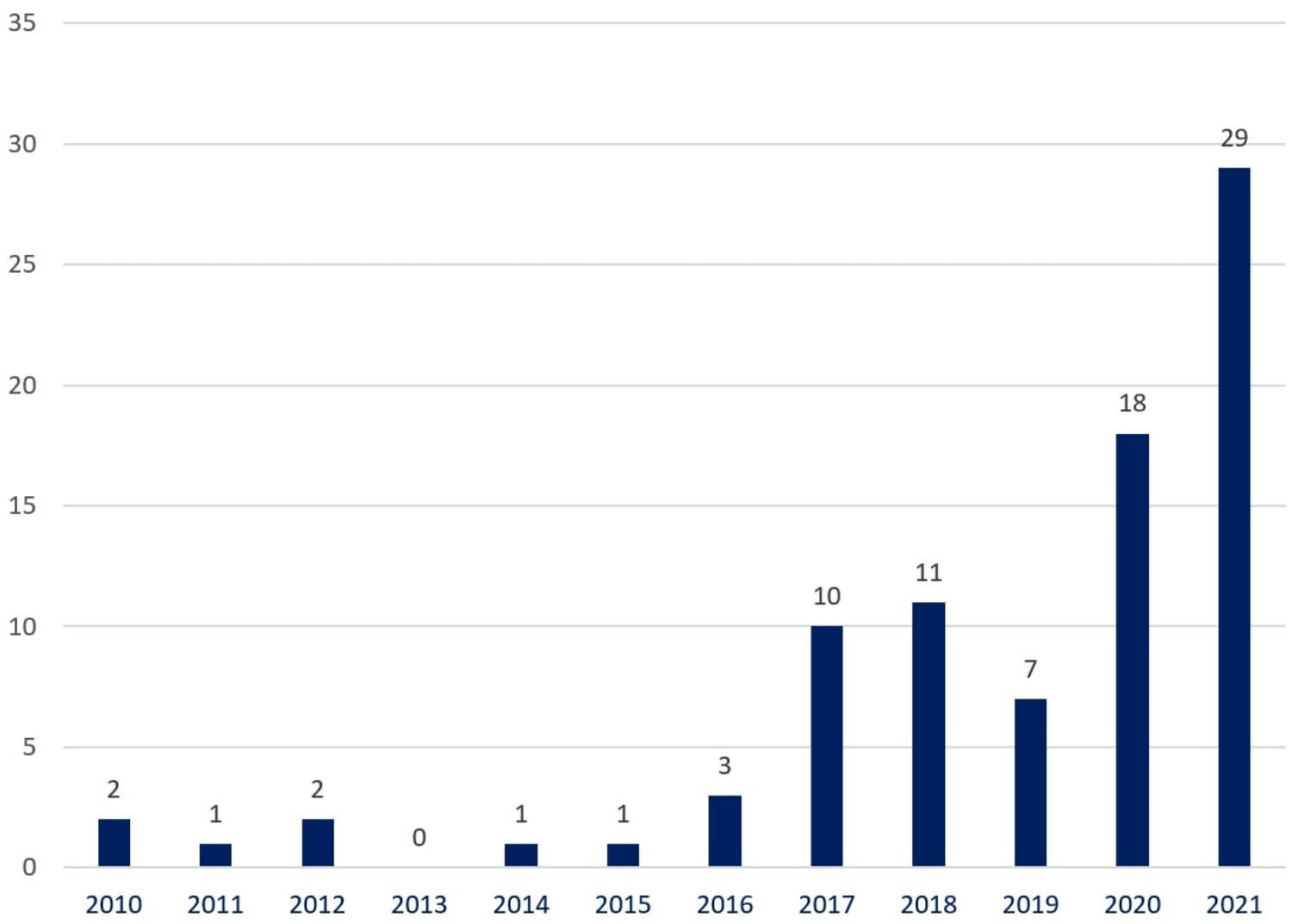
Number of publications per study year for the SLR

Concerning the publication frequency, the observation result shows that between 2010 and 2021, seven (7) studies were published per year on average. Figure 14 displays that the observed number of published studies was low until 2017. Since then, the number of published works has grown. Between 2020 and 2021, the number of publications has shown tremendous growth. This significant growing number of publications depicts that nowadays, web researchers are concerned about the importance of accessible web and ensuring accessibility of the digital platform.

**Fig. 15:**
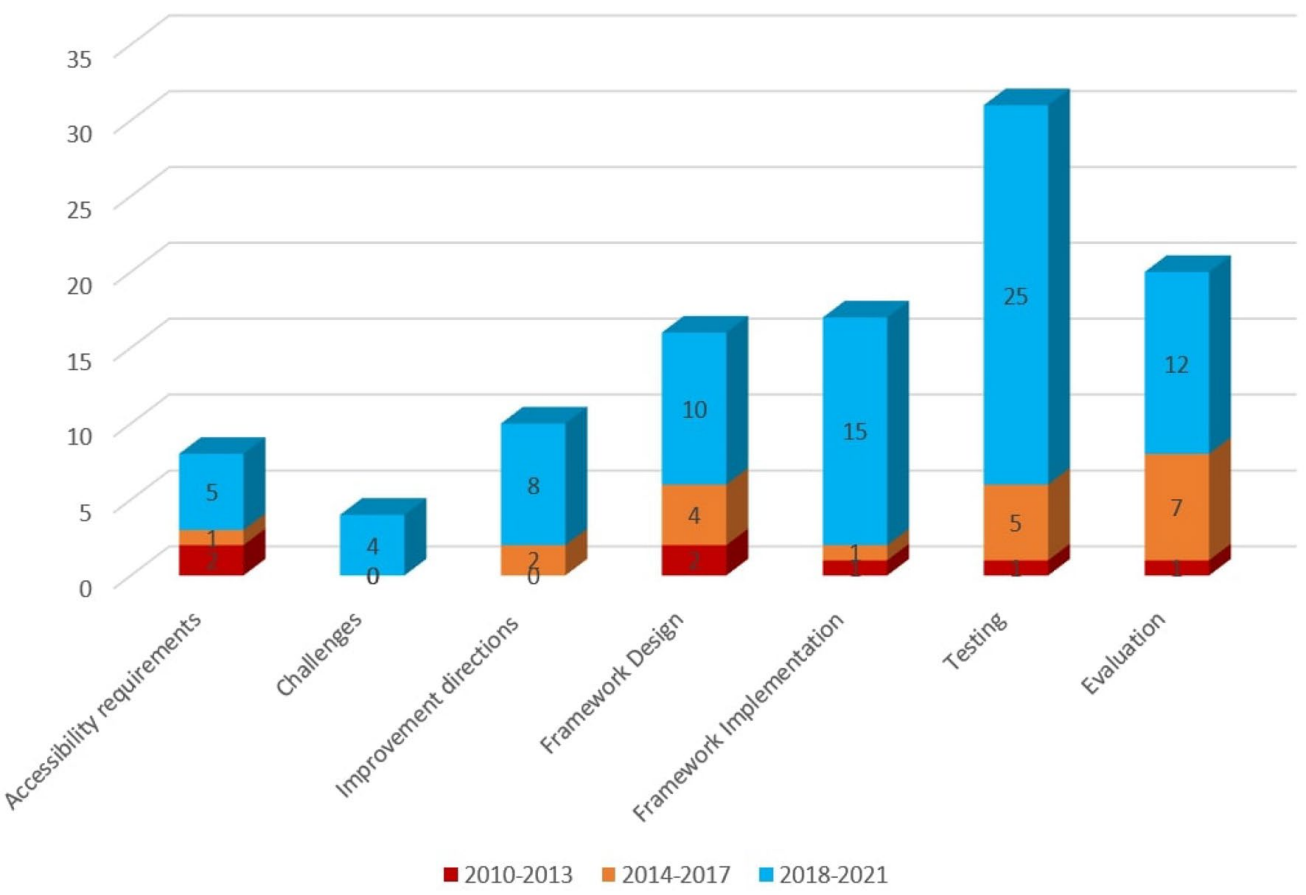
Number of publications in seven processes of the SLR considering three time periods

Considering our seven processes, we classified the selected papers into three periods: 2010–2013, 2014–2017, and 2018–2021. As shown in Fig. 15, the number of publications between 2018 and 2021 was much higher in each of the 7 processes compared to the earlier periods. This increase was greatest in testing. The rise of articles in the implementation, evaluation, and design areas is also remarkable. These statistics indicate that concern about digital accessibility has increased in recent years. Compared with other processes, accessibility requirements, challenges, and improvement directions are underrated topics in accessibility research. In addition, the number of papers for development methods (development and implementation) is also limited. This observation directs the importance of devoting continued efforts to conducting future research concerning accessibility requirements, challenges, improvement directions, and development methods.

**Fig. 16:**
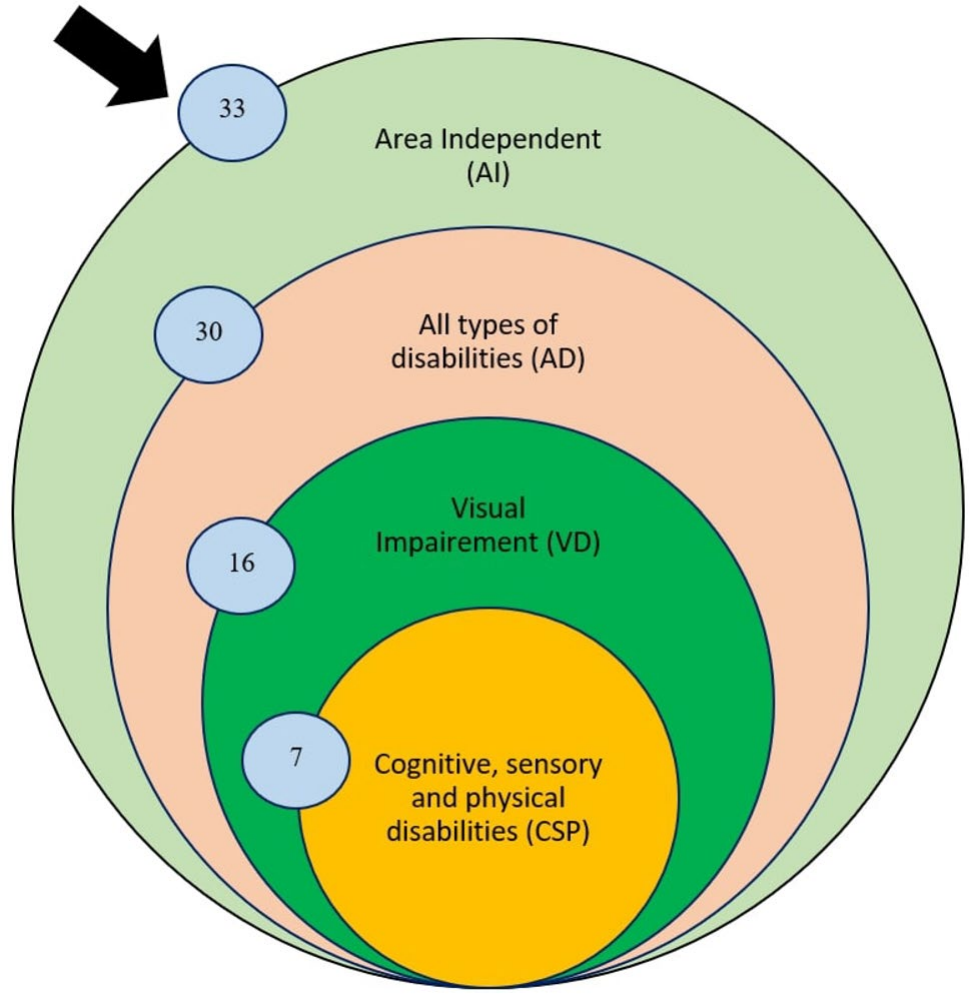
Publications with focused disabilities group

As the prime objective of accessibility research is to ensure online platforms are accessible to people with disabilities, thus, in this SLR study, we classified the past studies according to their focused disability type. Almost one-third of the selected studies did not focus on any group of disabilities (see Fig. 16). A prominent number of studies focused on issues with every disability. The number of studies focused on visual impairment is also noticeable. However, compared to these three criteria (AI (area independent), AD (all types of disabilities), and VD (visual impairment)), a few studies considered the cognitive, sensory impairment, and physical disabilities issues. Apart from the invested disability types, it is crucial to show the continued effort for other exceptional cases, such as hearing disabilities, moving disabilities, special children, and autism.

Despite the importance of applications to support during the web development process to ensure accessible application development, studies related to application development for accessibility direction are still limited compared to studies on web accessibility evaluation. This result shows the importance of putting effort into methods, tools, and assets to support the development of accessible web and web applications, considering the engineering feature of this platform.

### Web accessibility in past studies

In our search for past studies, we found seven SLRs addressing web accessibility. Najadat et al. [119] indicated that research on web accessibility has grown since 2007. However, the development of accessibility evaluation tools, metrics, and standards was addressed poorly by past literature. They showed the most common web metrics regarding design, speed, size, diagnosis tools, and metrics for better provision of services. Following this, an SLR carried out by Muniandy and Sulaiman [120] depicts that for years, accessible computer application design, including mobile applications, computer applications, and online web applications for visually impaired people, has gained immense popularity. Research conducted by Baldwin and Ching [121] identified that user-centric web prototype design would be helpful to improve accessibility in upcoming development for people with disabilities.

Addressing these issues, an SLR carried out by Akram and Sulaiman [14] indicated that many studies published between 2009 to 2017 devoted to automated tools development to validate the technical aspects against the accessibility conformance or guidelines. Despite the importance of automatic accessibility testing tools, the lack of advanced techniques to develop these tools required human observation to interact with people with disabilities with interactive systems. With the same focus, an SLR carried out by Campoverde-Molina et al. [15] stated that a synthesis study is crucial to determine the web accessibility standards and the evaluation methods. They also indicated that the testing process remains the main focus of the current web research. In another SLR, Campoverde-Molina et al. [16] added that the majority of the experimented websites have potential accessibility issues that address further investigation and more research in this field.

In our findings, we identified a few studies related to the accessible design pattern of rich internet application (RIA), accessibility guidelines visualization, and user interface designs. Compared with the previous SLR studies proposed by Akram and Sulaiman and Campoverde-Molina et al., our proposed study also identifies the importance and growth of accessibility requirements elicitation. They added that research on accessible development and evaluation techniques, user-centric design, and user requirements with disabilities should consider.

Further, an SLR conducted by Oh et al. [122] indicated that web accessibility research in the area of web image analysis and web-based gamification or game development has increased. They added that understanding visual information (e.g., images) is a critical challenge for people with low vision. Another SLR proposed by Salvador-Ullauri et al. [123] depicted that web-based games are helpful for teaching and learning for people with disabilities. Web and game developers and designers are fascinated by implementing accessible features as accessibility guidelines are not limited to a particular domain of people. However, from the comparative analysis of previous SLRs, we can observe that (Table 11) most of the past SLR studies have lacked consideration of development and implementation approaches for web evaluation that are necessary to include in our SLR process.

**Table 11:** Evaluation of past SLR considering the seven processes of the proposed SLR

References		Evaluation process						
No	Area	Accessibility requirements	Challenges	Improvement direction	Framework design	Implementation	Testing	Evaluation
Muniandy and Sulaiman [120]	Digital accessibility	✓(Yes)	✗ (No)	✗ (No)	✗ (No)	✗ (No)	✗ (No)	✗ (No)
Baldwin and Ching [121]	Digital accessibility	✓(Yes)	✗ (No)	✗ (No)	✗ (No)	✗ (No)	✗ (No)	✓(Yes)
Akram et al. [14]	Web	✗ (No)	✗ (No)	✗ (No)	✗ (No)	✗ (No)	✗ (No)	✓(Yes)
Campoverde-Molina et al. [15]	Web	✗ (No)	✗ (No)	✗ (No)	✗ (No)	✗ (No)	✓(Yes)	✓(Yes)
Campoverde-Molina et al. [16]	Web	✗ (No)	✗ (No)	✗ (No)	✗ (No)	✗ (No)	✓(Yes)	✓(Yes)
Oh et al. [122]	Web-based image	✗ (No)	✗ (No)	✗ (No)	✗ (No)	✗ (No)	✗ (No)	✓(Yes)
Salvador-Ullauri et al. [123]	Web-based game	✗ (No)	✓(Yes)	✓(Yes)	✓(Yes)	✓(Yes)	✗ (No)	✗ (No)
Proposed SLR	Web accessibility	✓(Yes)	✓(Yes)	✓(Yes)	✓(Yes)	✓(Yes)	✓(Yes)	✓(Yes)

### Observation of research

In the investigated studies of this research, among the considered seven processes, challenges, and accessibility requirements experienced with less literature. The primary reason might be aligned with the current research focus. The majority of the research focused on the development of evaluation and testing methods, though addressing accessibility challenges during web development and enhancing the importance of ensuring accessibility guidelines is also important [124]. Without demonstrating the challenges that might be raised during the development process and their associated solutions, it is barely possible to ensure accessibility for digital sources (e.g., websites, software, etc.). To improve these issues, more attention should be given to the current research focus to identify the major challenges associated with the development of the accessible solution and demonstrate the accessibility guidelines with its advancements. Besides, the literature for framework design and development/implementation is not significant compared to the other processes (e.g., testing). Also, there was limited investigation for evaluation metrics to evaluate the correlation between experimented results and user (e.g., people with disabilities) perceptions, which introduces an urgent need to investigate accessibility result validation systems. In addition, our SLR result illustrates that most of the research focused on automatic accessibility testing tools to investigate the accessibility of the web platform. The articles found considered automatic accessibility testing tools while largely neglecting engineering asset development. Therefore, our proposed SLR depicts the importance of future research for updated methods, techniques, processes, and approaches to support the ensurement of an accessible web.

However, a positive finding observed in this SLR was the rapid growth of the number of studies in the accessibility context. Improving accessibility means developing accessible applications and solutions to help users with various disabilities. This perspective emphasized that developed systems should focus on user requirements (especially for special needs users) to ensure user-centric design, considering user involvement and global accessibility design guidelines for digital inclusion. To enable accessible development tendencies in companies and governmental organizations, several governments have proposed rules to improve the accessibility of digital services; for instance, the United Kingdom, the European Union, the Chinese government, and other public and private organizations. Despite several new digital content accessibility guidelines, investigating new processes, tools and techniques is a significant challenge that directs the importance of future investigations or state-of-the-art research.

## Conclusion

A systematic literature review is presented in this paper, considering accessibility in the context of web evaluation processes. In this paper, we attempted to take a small step toward contributing to this research by pointing to a new direction for future goals and considerations.

This study showed automatic accessibility testing and evaluation of the focused area of research in the last decade for ensuring the inclusion of accessible web content. There was a great increase in the number of published works after 2017 compared to the previous years.

In the past, most of the literature focused on visual impairment, and very few papers discussed other disabilities, such as hearing, physical, and cognitive disabilities. In this SLR, we found requirements, challenges, engineering techniques, ontology, frameworks, API, algorithms, and testing tools for different levels of satisfaction associated with disabilities, but especially for visual impairment. Therefore, we identified and reported a research gap regarding other disabilities.

Unfortunately, there are few reference architectures for referring to accessible web design, development, and evaluation processes. For example, a framework for accessibility improvement of people with color vision deficiency [79], an approach for automatically identifying widgets [80], and an accessibility testing and refinement tool for the early design phase [110]. It would be beneficial to develop other reference architecture focusing on other contributing areas to solving three problems: (i) framework for the developer to identify and implement accessibility features to improve the accessibility issues, (ii) easy methods to understand and ensure accessibility requirements concerning every type of disabilities during the development phase, and (iii) updated automatic accessibility testing protocols incorporating the latest WCAG standards rules. To overcome these problems, we can note that developing new methods and tools could be a research topic in the upcoming years.

Considering the accessibility of current web platforms, in general, currently available web resources (websites, web-based games, web/mobile applications, etc.) are not accessible. Recently, the governments of many countries-imposed accessibility-related laws (i.e., WCAG) to ensure accessibility requirements. Furthermore, the methods and tools to solve the accessibility problems have limitations that direct future research concerning the development of engineering approaches.

For current accessibility research, there are many challenges to incorporating updated WCAG. Regarding automatic accessibility testing protocol, several studies focused on the limited number of guidelines and disability requirements. Studies for the design and development of accessibility testing protocols are limited. Thus, automatic accessibility testing protocol development concerning different disabilities and elderly user requirements could be a research area in the upcoming years.

Finally, consideration of several methodologies and open-source developments for ensuring accessibility is significantly important. Recently, several researchers and companies have been developing web-based solutions by adopting accessibility requirements. They develop open-source software that has an essential role for end-users and corporations. Accessibility is a crucial technological aspect of developing a new solution for any domain.
